# X-Ray Lithography Mask Metrology: Use of Transmitted Electrons in an SEM for Linewidth Measurement

**DOI:** 10.6028/jres.098.032

**Published:** 1993

**Authors:** Michael T. Postek, Jeremiah R. Lowney, Andras E. Vladar, William J. Keery, Egon Marx, Robert D. Larrabee

**Affiliations:** National Institute of Standards and Technology, Gaithersburg, MD 20899-0001

**Keywords:** lithography, metrology, secondary electron, SEM, transmitted electron, X ray

## Abstract

X-ray masks present a measurement object that is different from most other objects used in semiconductor processing because the support membrane is, by design, x-ray transparent. This characteristic can be used as an advantage in electron beam-based x-ray mask metrology since, depending upon the incident electron beam energies, substrate composition and substrate thickness, the membrane can also be essentially electron transparent. The areas of the mask where the absorber structures are located are essentially x-ray opaque, as well as electron opaque. This paper shows that excellent contrast and signal-to-noise levels can be obtained using the transmitted-electron signal for mask metrology rather than the more commonly collected secondary electron signal. Monte Carlo modeling of the transmitted electron signal was used to support this work in order to determine the optimum detector position and characteristics, as well as in determining the location of the edge in the image profile. The comparison between the data from the theoretically-modeled electron beam interaction and actual experimental data were shown to agree extremely well, particularly with regard to the wall slope characteristics of the structure. Therefore, the theory can be used to identify the location of the edge of the absorber line for linewidth measurement. This work provides one approach to improved x-ray mask linewidth metrology and a more precise edge location algorithm for measurement of feature sizes on x-ray masks in commercial instrumentation. This work also represents an initial step toward the first SEM-based accurate linewidth measurement standard from NIST, as well as providing a viable metrology for linewidth measurement instruments of x-ray masks for the lithography community.

## 1. Introduction

Optical and electrical linewidth measurements have been the traditional methods of monitoring and controlling microelectronics fabrication processes. As the feature sizes in these processes approached and ultimately became smaller than the wavelength of the light used in the optical measurements, the industry turned to the scanning electron microscope (SEM) for this monitoring. The SEM solved some problems limiting accuracy in linewidth metrology, but also introduced some new problems of its own [1,2]. This paper describes a nonconventional transmission-mode of the SEM as applied to x-ray masks that reduces the impact of some of the metrological problems of accurate linewidth metrology in the SEM. However, at the present time, there are few process control applications within the semiconductor industry where the transmission electron detection mode can be used. Many of the concepts described here can be eventually employed in these other applications since the concepts of the transmitted electron detection (TED) mode form the basis for future accurate backscattered and secondary electron metrology. The utility of this mode for metrology arises from the relative insensitivity to the inevitable approximations that must be made in the modeling of electron scattering and detection required for accurate edge detection [3,4]. The present work should be of particular interest to the SEM metrologist who is concerned with either feature size measurement or the detection of defects in the x-ray mask structure and contamination on its surface. The utility for inspection arises from the fact that the transmission of electrons through the mask for lithography is, in some ways, analogous to the transmission of x rays through the mask.

Perhaps the three most important unsolved measurement issues in controlling the processes of fabricating integrated circuits by optical lithography are; 1) accurate measurement of linewidth, 2) detection and characterization of geometrical faults, and 3) measurement of mask-to-mask pattern placement errors. The same three issues will undoubtedly be important in x-ray lithography. This paper addresses the problem of measuring linewidth and line spacing (i.e., pitch) on x-ray masks by making use of the transmission electron detection mode of the scanning electron microscope [4,5,6] referred to as the transmitted scanning electron detection mode (TSEM). This technique utilizes a TED system which is mounted below the sample. The application of TSEM is made possible by the presence of a relatively thin supporting membrane composing these masks which is essentially electron transparent for beam energies as low as about 15 keV (for the present samples and detector arrangement). The TSEM mode is also useful for inspecting x-ray masks for defects because, to a first approximation, the electrons interact with the mask in much the same way as the X rays do in producing their transmission-mode image. For example, defects such as a small void inside an x-ray absorber line will be seen in transmission by both x rays and electrons. However, they will not be seen by visible light, perhaps not by conventional secondary-electron SEM imaging, and certainly not by scanning probe imaging.

X-ray lithography process engineers want accurate dimensional measurements, but accuracy is an elusive concept that everyone would like to deal with by simply calibrating their measurement system by using a NIST developed standard. Unfortunately, it is not easy either for NIST to calibrate submicrometer standards or for the engineer to use standards in calibrating instruments. Accurate feature-size measurements require accurate determination of the left and right edges of the feature being measured. The determination of edge location presents difficulties for all current measurement technologies because x-ray mask features are generally not thin compared to the wavelength of light in the optical microscope [1], or the electron scattering range in the SEM [2], or the size of the probe in scanning probe systems such as the atomic force microscope [7]. Since linewidth is a left-edge-to-right-edge measurement, an error in absolute edge position in the microscopic image of an amount Δ*L* will give rise to an error in linewidth of 2 Δ*L*. If any technique could be found that produces a step-function response at the location of the geometric edge in its image, there would be no problem in identifying that edge position. However, to date, no such technique has been found. This paper demonstrates that the TSEM images can have a relatively rapidly changing intensity in the vicinity of the true edge position and, therefore, can be made inherently less sensitive than the conventional SEM modes to this source of error in linewidth measurements. The TSEM technique is not inherently more or less accurate than other SEM modes for pitch measurements because pitch measurements are not subject to this type of error (if the two lines in question have similarly shaped left and right edges). Therefore, the present paper will concentrate on TSEM-based linewidth measurements.

Conventional transmission-electron-mode (TEM) operation of a scanning electron microscope utilizes an electron detector with a very narrow cone-angle of acceptance designed to detect only the unscattered component of the transmitted electron beam. An alternative would be the dark-field mode where only the scattered electrons are collected. The reverse is true for the present TSEM technique where a broad acceptance angle detector is used to detect as many of the transmitted electrons as possible (i.e., whether scattered or not) that have an energy above some predetermined threshold which is usually several keV. Then, the electrons are physically filtered both by the signal threshold characteristics of the detector *and* a physical filter in front of the detector. This greatly improves the contrast level over the conventional TEM mode for this type of application, and greatly simplifies the required Monte Carlo modeling. It is, in fact, this change in electron detection philosophy (compared to more conventional TEM operation) that makes the present TSEM approach so attractive for dimensional metrology and inspection of x-ray masks. Therefore, the TSEM mode should be attractive to both NIST (for use in calibrating future x-ray mask standards) and the x-ray lithographic community (for use in metrology instruments to minimize inherent sources of inaccuracy).

The results of measuring the widths of nominal 0.25, 0.35, 0.5, and 0.75 μm wide lines and pitch arrays on actual x-ray masks with the TSEM mode of operation of the NIST Metrology SEM [8] are presented. The results of Monte Carlo simulations of these measurements are also presented and compared to the experimental data. These Monte Carlo results predicted a definite characteristic notch in the transmission-mode image profile as the electron beam traverses the sloping edges of an ideal trapezoidal shaped line. The measured lines actually were, to a first approximation, trapezoidal but with rough sloping surfaces that made observation of the notch difficult, but not impossible. This paper reports on the theoretical existence of this notch, its observation, and discusses metrological significance that makes the TSEM mode even more attractive than indicated above.

The x-ray mask work presented here represents one method under development at NIST to measure, and ultimately certify x-ray mask linewidth standards for the x-ray lithography community when the interest and need arises. However, the use of the TSEM mode is also recommended for use by that community for its routine metrology, inspection and repair of x-ray masks.

## 2. Metrology Issues and Implementation

### 2.1 X-Ray Masks

**Fig. 1:**
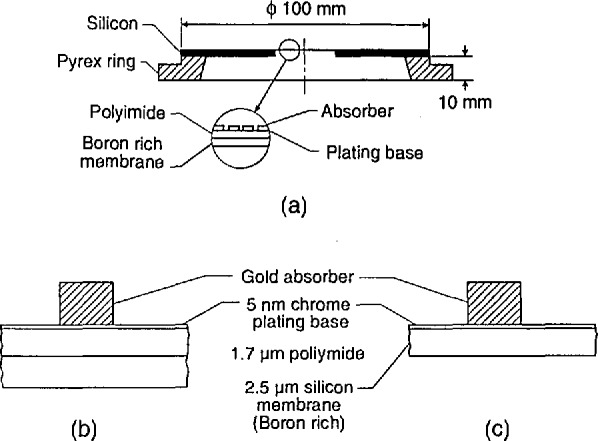
Diagram showing an enlarged cross section of the x-ray mask studied in this work, (a) View of the entire mask assembly. (b) Diagram of the original membrane-absorber cross section. (c) Diagram of the modified version without the polyimide.

**Fig. 2:**
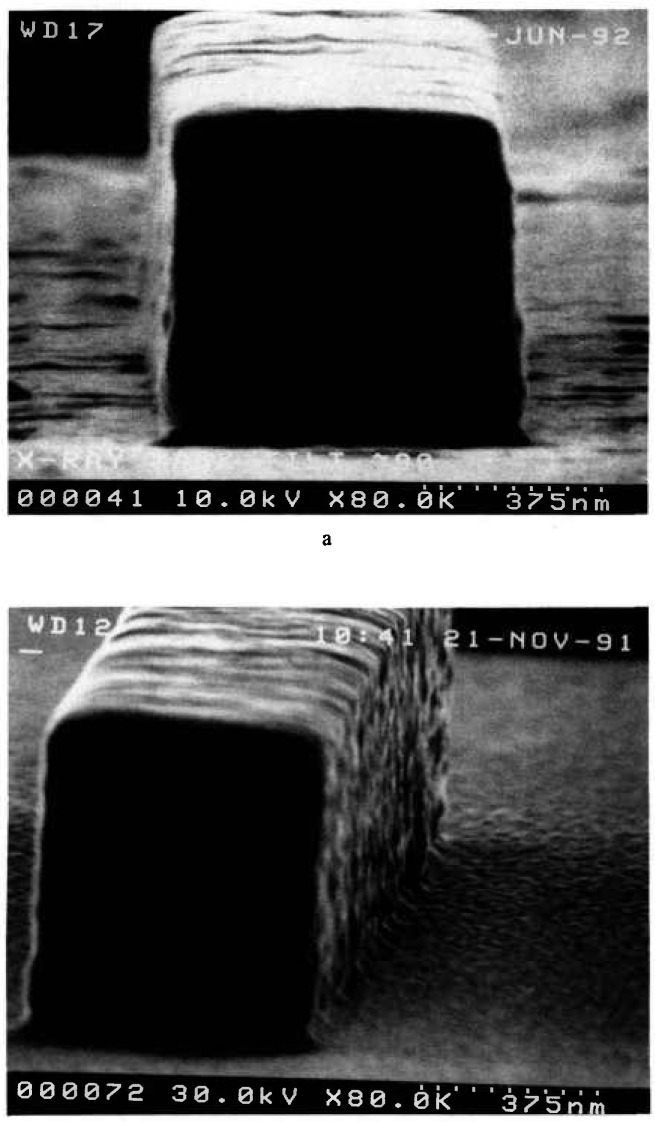
Scanning electron micrographs of the gold absorber lines at high tilt, (a) High tilt nearing a cross sectional view. Note the edge roughness and irregularity, (b) Micrograph similar to (a) but including some sample rotation in order to further observe the sidewall structure.

Masks utilized in x-ray lithography are of various constructions depending upon the manufacturing process employed. However, except for the thickness (affecting electron transparency) of the supporting membrane and chromium coating, this variability should have little bearing on the present technique. Test masks for this study were obtained from IBM Corporation^1^ [9]. A drawing of the overall mask can be seen in Fig. 1a. The masks are composed of a support membrane of approximately 2.5 μm of boron-doped silicon upon which 1.7 μm of polyimide is placed (Fig. 1b); other masks manufactured later in this work had the polyimide layer removed (Fig. 1c). The absorber structure was patterned in a layer of gold approximately 0.7 μm thick over a 5 nm chromium plating base. Other types and designs of x-ray masks available from other manufacturers that differ somewhat from these figures are not addressed in this work. The patterned features of the test mask used for this work have some edge irregularities (Fig. 2a) which limit the measurement precision and increase the measurement uncertainty. The edges of the absorber are slightly rounded and are reasonably vertical in cross section but have a slight slope (discussed later) which can also influence edge detection (Fig. 2b).

### 2.2 Scanning Electron Microscopes

Imaging and measurements for this work were done by using the NIST metrology instrument based on an AMRAY 1610 scanning electron microscope equipped with a lanthanum hexaboride filament. Supplemental imaging and beam-scanned metrology was also done by using a field emission Hitachi S-4000 scanning electron microscope (FESEM).

#### 2.2.1 NIST Metrology Instrument

The fundamental construction aspects of the NIST metrology instrument have been published previously [8]. This instrument, as most standard laboratory scanning electron microscopes, was factory-equipped with a maximum 30 kV accelerating voltage. This system was later modified to have a computer-controlled, cathode-stabilized 30 kV power supply system [10]. One important experiment performed with the instrument was to retrofit in the field a 50 kV accelerating voltage power supply system, in order to test the potential advantages (and disadvantages) afforded by the higher accelerating voltage on TED metrology, imaging, and resolution on the x-ray masks. Most typical SEMs operate at or below 30 kV accelerating voltage but other types of electron beam instrumentation, notably the typical scanning transmission electron microscopes (STEM) can operate at much higher accelerating voltages. The comparison work (between 30 and 50 kV), is discussed below and in our earlier work [4]. This work demonstrated that there was no advantage in doing the metrology at accelerating voltages over 30 kV for the design of x-ray masks investigated in this work. This means that most standard commercial SEMs can be converted to x-ray mask metrology systems with no major instrument modifications. Therefore, the NIST metrology instrument was returned to the computer-controlled 30 kV configuration.

#### 2.2.2 Fundamental Metrology Instrument Operation

In operation, the sample area to be measured is visually positioned by using the standard raster scan. Then the electron beam of the metrology instrument is “fixed” in position by switching into spot mode. The electron beam acts as the reference point for the measurement. The philosophy behind this technique has been discussed previously [11]. The object to be measured is then translated beneath the electron beam by an electromechanically-scanned stage [12,13]. The linear displacement of the stage is precisely determined by use of a commercial optical interferometric measurement system. As the sample is scanned, the collected electron signal is stored simultaneously with the data from the interferometer system by a dedicated microcomputer system. The output of the system is a graph of the measured transmitted signal plotted against the interferometrically determined scan position (i.e., the image profile). Subsequent analysis of this image profile and its comparison to the profiles determined from the Monte Carlo modeling determine the location of, and the distance between, the left and right edges of the line being measured.

#### 2.2.3 Laser Interferometer Stage

A precise laser-interferometer piezo-flex stage, custom fitted to the SEM chamber, was developed in order to accurately monitor the specimen motion to within a few nanometers under computer control. The original stage [8] was modified extensively for the measurements of the x-ray masks. These modifications were necessary to facilitate the large x-ray mask reticle and pyrex support ring as well as the transmitted-electron detector scheme employed for this work. This mask was far larger than any sample originally envisioned for measurement in this instrument. The metrology stage used in this instrument is similar in principle, but not in practice, to the stage used in the other NIST metrology instrument currently used to certify the SEM magnification standard SRM 484 [14]. The total range of travel of the present piezo-flex stage is limited to about 70 μm, but this can be circumvented by the 100 mm range of coarse stage. Therefore, the larger samples generally associated with the semiconductor community can be accommodated as long as the area of interest is positioned in the center of the wafer or mask. Limited fine-positioning motion is available in both the X and Y directions. Currently the maximum measurement distance is limited by the maximum piezo stage travel although staging and software changes are planned to extend this motion to include the mechanical stage motion.

**Fig. 3:**
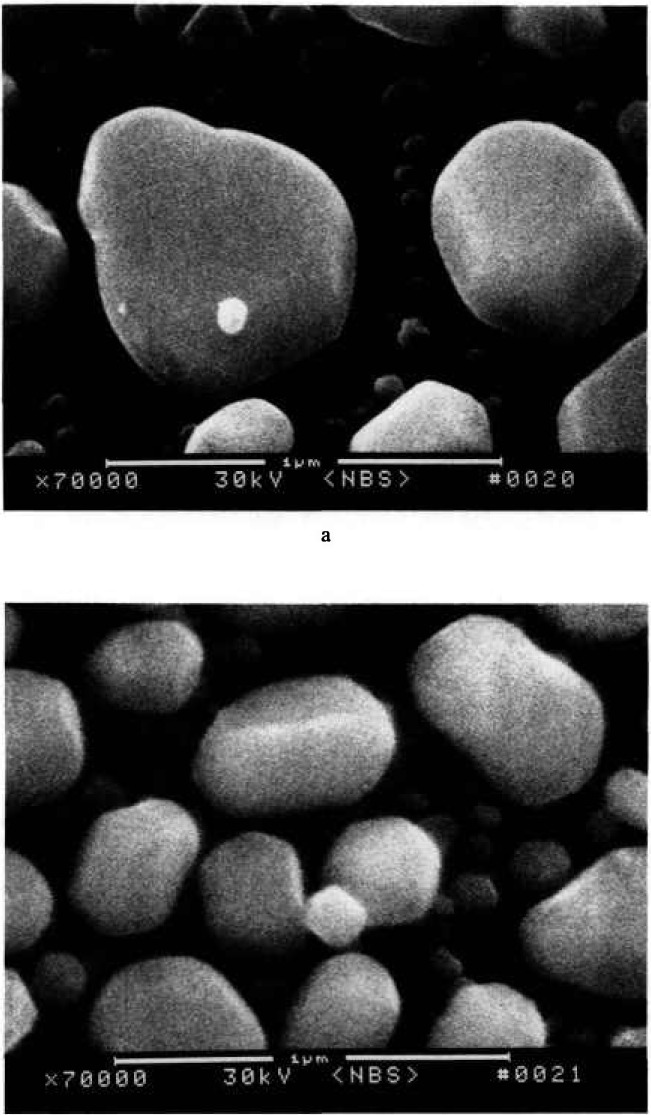
Scanning electron micrographs of a standard gold-on-carbon resolution sample using the NIST LaB_6_ metrology instrument, (a) Short working distance (i.e., 4 mm) and (b) long working distance (i.e., 12 mm) where the sample on the laser interferometer stage resides. Note the loss of sharpness in the long working distance micrograph.

The laser interferometer stage is designed so that the sample resides at a fixed 12 mm working distance below the final lens polepiece of the instrument. This long working distance has the unfortunate effect of limiting the resolution of the instrument from the specified and demonstrated 4 nm resolution to about 10 to 15 nm ultimate resolution under these conditions. The effect of the working distance on image sharpness is shown in Fig. 3 by using a standard gold-on-carbon resolution specimen. The only major instrument parameter changed between the two micrographs was the working distance. The image was then refocused and re-astigmated. These micrographs demonstrate that the reduction in sharpness is directly related to the increased working distance.

The stage motion is tracked by optical laser interferometry. The interferometers for both the X and Y measurements are dual-pass plane mirror (quad-beam) Michelson-type interferometers [15], with a least-count of 2.5 nm. The interferometer is mounted directly in the vacuum chamber in order to minimize both the dead-path and any environmental influences. The laser source is a Zeeman-stabilized He-Ne laser which emits reference and measurement beams of orthogonal linear polarization separated by a frequency of about 2 MHz. The mirrors reflecting the laser beam back are mounted in X and Y above the piezo stage in direct line with the sample. This position minimizes any Abbé-off-set errors. Displacement of the mirrors in either the X or the Y directions appears as phase information on a radio-frequency carrier and is detected by standard heterodyne techniques. The entire laser-interferometer stage unit is composed of two joined sections: 1) the laser, the directing optics and the receivers which are all external to the vacuum; and 2) the interferometer optics, stage assembly and sample which are all in the vacuum space of the electron microscope. The entire stage is removable from the vacuum as a unit in order to facilitate all alignments before being installed within the microscope chamber. All the adjustments have locks so that once the stage has been placed into the vacuum no change in alignment occurs.

Prior to the x-ray mask work, the laser-interferometer metrology stage was designed around secondary and backscattered electron detection and measurement by using a microchannel-plate electron detection system [16]. As part of the extensive modifications to the stage for the x-ray mask metrology, a TED facility was also included as described below.

#### 2.2.4 Stage Control and Data Acquisition Software

The stage control for positioning and data acquisition is fully controlled by a Hewlett Packard model 320 microcomputer system connected through a Hewlett Packard model 3852 data acquisition system. This system is programmed to generate a stair-stepped voltage ramp to drive the piezo-flex stage and move the sample area under the stationary SEM beam. At the highest of the four resolution settings available, the system is capable of three to four thousand steps per micrometer of travel. After the operator’s choices have been made, such as direction and length of scan, the sample is visually positioned and then the scan is started and measurement data are taken. A count from the laser system electronics and a voltage reading from the electron detector electronics are taken simultaneously at each step of the ramp and stored by the data acquisition system. At the completion of the scan, all data are transferred to the microcomputer and the raw laser counts are converted to micrometers. This original set of data pairs may be graphically displayed on the computer for review.

The construction of the piezo stage coupled with the relatively heavy assembly of mirrors, sample holder, and sample which it carries, causes it to be subject to some vibration effects, especially during sample motion. Considerable effort went into reducing the effects due to external sources of vibration, and measurements confirm that these are held to plus or minus one laser count (i.e., 2.5 nm). However, under stage drive, there is a vibrational motion superimposed onto the desired linear motion. The resulting plot of voltage signal versus laser position is not a smooth curve. This is not a serious concern as long as the data for the image profile are made simultaneously and reflect the position and signal at the same time. A method of smoothing the raw data and reducing the total amount of data to be analyzed was adopted. The data were first sorted by increasing value of laser reading (position) and then multiple voltage values (signal intensity) at each different laser reading were averaged and replaced by this single value. This is equivalent to making several readings at a single point and then reporting only the average as a means of increasing the apparent signal-to-noise ratio. In practice, this means that a raw scan of over 20 000 data points over several micrometers (such as in a pitch measurement) is reduced to about 2 000 data points by this procedure. Both the original and this reduced data set may be printed, stored for future use, recorded on floppy disk, or transmitted to a larger computer for analysis and computation.

A second data measurement program for the computer has been developed [17] and is still undergoing further development. This program, in addition to providing for control of the stepper motor driven stages, can read the stored data files and display the resultant curves. On these plots, the regions between movable gates may be expanded to show line edges in greater detail. Cursors may be moved along the curve with a display of the laser position and corresponding voltage shown below the plot. With this program, the edges of the feature being measured are not determined subjectively by moving the cursors over the displayed image profile (as done by most current measurement algorithms), but by the results of the modeling of the measured x-ray masks as described below. Using the model-derived edge criterion and the plots in this program, edge locations were manually determined and appropriate widths and pitches reported. Future modifications to this program will facilitate automated operation.

### 2.3 SEMs and Data Acquisition

#### 2.3.1 AMRAY Measurement Systems

Comparison measurements were made of TED mode image profiles from the digitally-acquired video data from two sources: 1) the commercial AMRAY beam scan linewidth measurement system accurately calibrated to NIST SRM 484, and 2) the laser interferometer stage described above. Since the transmitted electron detection system can be common between the two measurement systems, comparison measurements can be made with little difficulty. However, the pixel resolution between the two systems differs significantly.

#### 2.3.2 Hitachi Measurement System

The same sample holder and TED system (described in Sec. 2.4.2) was carefully inserted into the Hitachi S-4000 field emission instrument. Only slight modifications to the amplification system were required. The Hitachi field emission SEM was also equipped with the Hitachi keyboard measurement system accessory and was accurately calibrated by using NIST SRM 484 at high accelerating voltage. This instrument was also useful in the determination of the edge precision of the absorber lines at high magnification and resolution on broken mask pieces. It was also used to measure the wall angle. But, since these data are unable to be directly transferred to an ancillary computer system for image analysis, precise measurements could not be obtained directly from this instrument and a secondary system described below was developed.

#### 2.3.3 ISAAC Image Analysis System

A computer-based measurement system christened “Isaac” was developed for analysis of the digital images from the Hitachi S-4000 SEM or other video input [18]. The images are captured with a high-speed frame-grabber called a PIXEL PIPELINE [19]. The software generally used on the system is a commercially available image analysis program called IP Lab Spectrum [20] and also the public domain program named, “Image” of the National Institutes of Health [21]. The display rate is 30 frames/s (real time video on the computer screen), the spatial resolution is 640 × 480 pixels with 256 gray levels. Further improvements of this system for high resolution digitization (4048 × 4048) have also been implemented.

The video signal for the Isaac system was acquired at TV rates or scanned into the computer using the scanner and then stored and processed in the computer. The pixels of both the scanner and the Isaac have been calibrated with precise linear scales. With the Image and the IP Lab Spectrum Programs, there is the capability to control the frame-grabber card, and the capability to use the built-in tools, modifications, pseudocolorization, calculations, and measurements. The IP Lab Spectrum program also has an extension developed by Signal Analytics [20] in collaboration with NIST specially designed for linewidth or pitch measurements used in this work.

### 2.4 Electron Detection System

The development of the electron detection system used in this work was divided into three parts. The first was the electron scattering modeling used to study the geometry of the TED detection system. The second was the experimentation necessary to compare different detection systems available and adaptable for use. The third was to determine the optimum conditions for the measurements.

#### 2.4.1 Geometry of the Electron-Detector

**Fig. 4:**
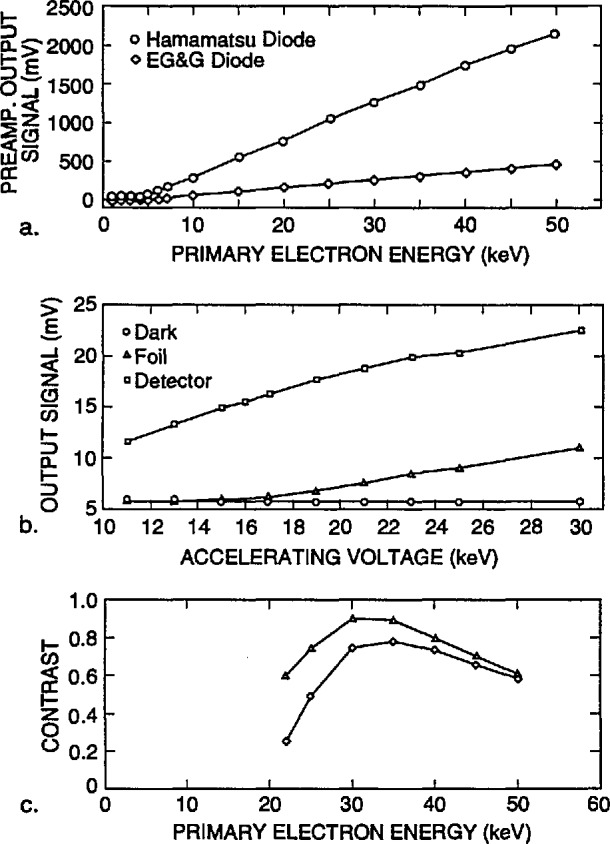
Semiconductor diode detector, (a) Comparison of the cut-off energy for two types of diodes tested and used as transmission electron detectors. Note the cut-off energy is at about 5 keV. (b) Comparison of the unfiltered signal of the transmission electron detector to the same detector equipped with a thin copper foil filter. Note the minimum energy reaching the detector to generate signal is now about 16 keV. (c) Comparison of the response of two detector types within the range of 20–50 keV. Note that the contrast peaks in about the 30–35 keV region.

System Monte Carlo modeling of the transmitted-electron signal showed that the angle subtended by the detector should be maximized to obtain a large enough signal with good edge contrast. This required the development of a special detection system, a highly-efficient transmitted electron detector, and high-gain and low-noise amplifiers that would fit within the limited space available in the instrument. A semiconductor diode detector was selected for this that had a threshold detection energy of about 3–5 keV (Fig. 4a) and therefore was insensitive to the secondary or low-energy transmitted electrons. This insensitivity was an advantage for this work. The main causes of this insensitivity are a gold surface barrier (the ohmic contact) on the diode and a thin dead layer above the real detecting p-n junction. Therefore, those relatively low-energy electrons having undergone high-energy loss because of inelastic collisions, but with a trajectory leading to the detector, are filtered from the measured signal. In addition, placement of electron barriers such as pre-filtration grids or metallic foils can reduce the highly-scattered fraction even more. A very thin (approximately 0.5 μm) copper foil was placed above the detector ensuring that the electrons having less than about 10 keV energy are not detected (Fig. 4b). Since the number of electrons reaching the transmitted electron detector through the filter is rather small, in this particular application, the gain and performance of the detector/amplification system must be high.

#### 2.4.2 Selection of the Semiconductor Diode Detector

The first detector used as a transmitted electron detector for this work, was a HUV 4000B photo diode/operational amplifier combination [22]. The HUV 4000B, consists of an approximately 11 mm outside diameter silicon diode with an active area of 100 mm which was ordered without the passivation layer to maximize the sensitivity. This diode has a built-in FET first-stage amplifier with a feedback resistor. The frequency range is DC - 0.1 MHz (gain bandwidth product). This detector proved to be adequate to demonstrate the utility of the transmitted electron collection and evaluation of the technique. Early experimental work was done using this detection system. Later, a new detector/amplifier was designed and assembled with a discrete detector and preamplifier [23]. This detector, a S3590-02 type, PIN silicon photo diode was specially advantageous because it has large, 10 × 10 mm photon sensitive and thus electron sensitive area. Two preamplifier stages are also mounted at the detector, consisting of two OPA 637 BP type integrated circuits [24]. This design showed better signal-to-noise ratio and wider bandwidth than the previous design (Fig. 4c), allowing it to work with picoampere primary beam current, therefore permitting the condenser lens smallest spot size setting and high non-TV scan rate. In the design of the detector/amplifier, it was important to take into account the following considerations (as well as those discussed above):

1. *Available Space*. There is a relatively narrow (11 mm) space underneath the x-ray mask where a detector could be placed, (Fig. 1a).
2. *Weight of the detector amplifier assembly*. The total weight of the assembly must be as small as possible and the wiring used must be as flexible as possible to minimize any vibration transmitted to the stage assembly.
3. *Location Flexibility*. The detector itself should be easily moveable underneath the mask to be able to place the most sensitive area in the correct location beneath the pattern.
4. *Power Consumption*. The detector/amplifier should be a low-power type to eliminate any possible heat dissipation.
5. *Cleanliness*. The design must meet the requirement of cleanliness and high-vacuum compatibility.

**Fig. 5:**
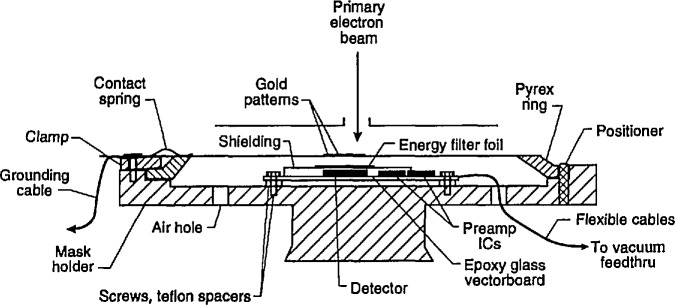
Cross section diagram of the detector/mask holder assembly developed for the NIST metrology instrument.

The sample holder/detector design as shown in Fig. 5 fulfills the necessary requirements for this detector assembly and is currently in use. All the measurement work was done with this detector although some of the experimental work shown may have been done with the other experimental designs.

The detector/amplifier assembly is constructed on lightweight Vector board. This board is an epoxy glass panel having holes in a raster of a tenth of an inch that it is easy to position and mount the components on it and to place the detector to the required position. The ICs are powered with only ± 6 V to minimize the power consumption and thus heat dissipation. The detector and the integrated circuits are in thin, special, gold-plated sockets and the components are soldered on the back side. After the final assembly, the circuitry was carefully cleaned and dried and the detector and the active elements were inserted in place. The detector has no passivation layer and is kept in vacuum so that it remains clean and free of any noisecausing moisture or dirt.

The wiring is made of a specially-flexible shielded cable similar to that used in record turntable tonearms. These wires are flexible enough not to transmit disturbing mechanical forces to the stage and do not act as small springs. The cables terminate in plug-in receptacles allowing simple mounting and removal for sample exchange. When the mask holder is in place, fastened on the piezo-electrical stage and the cables are connected, the assembly is ready for operation.

#### 2.4.3 Optimal Conditions

The third part of this study was to find the suitable, optimal conditions for the measurement. Beyond the obvious choices like the small spot size for good resolution, the parameters of bandwidth, signal conditioning to improve the signal-to-noise ratio and the optimum of energy filtering had to be found. Fortunately, since the laser interferometer stage information is taken slowly, the bandwidth of the amplification system does not contribute to the measurement. The contrast distributions established experimentally show the best accelerating voltage region of the primary electron beam to be in the range of 20–30 keV, the maximum necessary, in this case, being about 30 keV. Figure 4c shows the ratio of signals on the gold stripes and on the membrane for the two different detector/amplifiers demonstrating a peak in this range. Thus, the metrology instrument was operated and the measurements made at 30 keV accelerating voltage.

### 2.5 Monte Carlo Electron Beam Modeling

Electron beam modeling done on the NIST Cray super computer was used to support the experimental work in several areas of this study. This work is used to compare experimental and theoretical results in order to suggest new approaches for the measurements, and to help in the interpretation of the experimental results. The Monte Carlo code used in this work produces predictions of the number of backscattered, secondary, transmitted, and filtered transmitted electrons as a function of the location of the incident electron beam from the simulated trajectories of electrons. Only the high-energy (filtered) transmitted electron data is of interest for this work. Input variables include: the electron energy, electron beam diameter, location of the incident beam on the sample relative to the feature of interest, the thickness of the component layers of the x-ray mask, and the characteristics of the solid-state diode detector.

The Monte Carlo computer code was based on the code generated by D. Newbury and R. Myklebust of NIST [25] based on the earlier work by Curgenven and Duncumb [26]. This code was modified for this work to model the particular configuration of the x-ray mask and to gather statistics about the electrons that are collected by the detector. This computer model has been used to determine the transmission of electrons through lithographically-produced gold lines on silicon substrates. A thin (5 nm) chrome layer, which improves the interface properties between the gold and the silicon, has been included. The Monte Carlo code is based on a screened Rutherford model for the atomic scattering. The scattering cross sections have been multiplied by the factor (1+Z/300), where Z is the atomic number, in order to improve agreement with measured backscattering coefficients. This factor is a zero-th order way to account for the differences between the screened Rutherford [27] and the more exact Mott cross sections [28]. A low energy cut-off has been included in the transmission coefficient because a filter is placed in front of the detector that eliminates electrons with energies below a known value (e.g., 16 keV). A total of 20 000 trajectories has been used in these calculations to reduce the level of error to less than 1%. The code allows the user to follow closely the changes in the number of transmitted electrons as the simulated electron beam traverses the mask from the substrate to the gold absorber strip. This modeling effort is currently being extended to include other concerns, such as the proximity effects of neighboring lines, detector geometry, detector sensitivity, and other geometric effects.

The Monte Carlo code was specifically modified to solve for the special case of the x-ray mask, that is, the transmission of electrons through gold absorber lines on thin silicon membranes. In this work, a gold line is modeled as a strip with trapezoidal cross section on an otherwise uniform plane. If an electron crosses the boundary of the line, then a backward step must be taken so that the previous step terminates at the intersection with that boundary. Energy losses are thus computed only up to this point. The calculations then proceed in the new layer (or in vacuum) until a predetermined stopping condition is reached. When the energy of an electron falls below a preassigned minimum value it is removed from further consideration. The energy losses are determined from the theory of Bethe [29]. Note that the specific energy losses due to secondaries are included in the Bethe formula and are not subtracted from the primary electrons, which would constitute double counting. The number of backscattered, secondary, and transmitted electrons are determined with the inclusion of a possible energy filter associated with the electron detector. The results are then printed as a function of the position of the incoming electron beam.

**Fig. 6:**
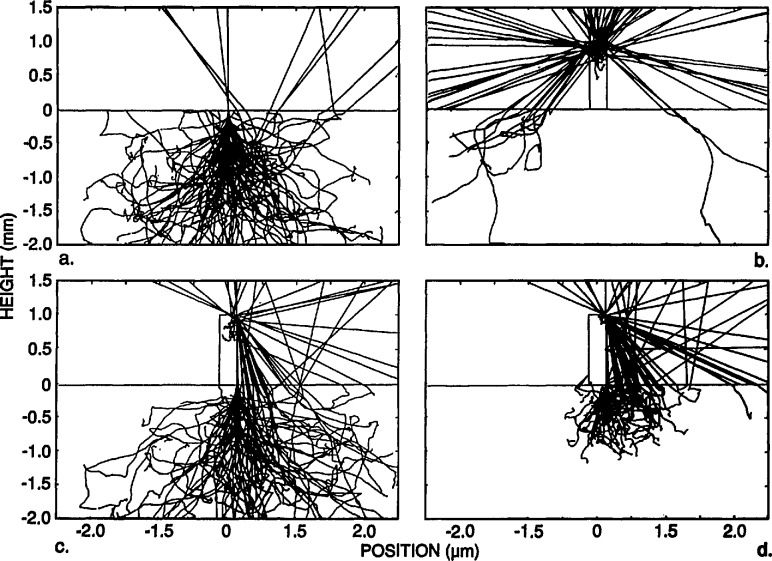
Monte Carlo modeling of the x-ray mask. (a) Simulated behavior of the electron beam incident on the membrane region. The primary electron beam is incident at the 0,0 position of the graph. Electrons leaving the membrane at height −2.0 are not shown in the figure, but pass on to the electron detector, (b) Simulated behavior of the electron beam incident on the center of the absorber structure, (c) Simulated behavior of the electron beam incident at the edge of the absorber structure (20 keV electron beam accelerating voltage and a vertical wall absorber), (d) Comparison of the simulated behavior of the electron beam incident at the edge of the absorber, but at 10 keV accelerating voltage where there is no electron transmission.

The primary emphasis of this work is to model the electron transmission near the edge of gold lines in order to identify the edge locations and thereby obtain a value for the linewidths. The transmission varies greatly as the electron beam crosses the edge. In principle, the edge may be found by superimposing the normalized measured and calculated transmission-electron image profiles and, if they agree with each other, the edge locations in the model profile are taken as the real edge locations. The model was used to simulate the behavior of 100 electrons for different incident locations on the x-ray mask as shown in Fig. 6. The incident electrons in this case are modeled to have an energy of 20 keV and a Gaussian spatial distribution with a diameter of 2 nm. The gold structure is modeled with a perfectly vertical wall. The electron beam in Fig. 6a is shown incident on the membrane far away from the gold absorber strip; in Fig. 6b the electron beam is shown incident at the center of the gold absorber strip; and in Fig. 6c the beam is shown incident at the edge of the absorber. A comparison of the electron behavior for 10 keV is shown in Fig. 6d where no electrons are transmitted. The figures shown are limited to a field width of 5 μm, although electrons are actually followed by the model out to a much larger distance. These electron trajectory plots illustrate the degree of electron scatter as the electron beam traverses the mask.

**Fig. 7:**
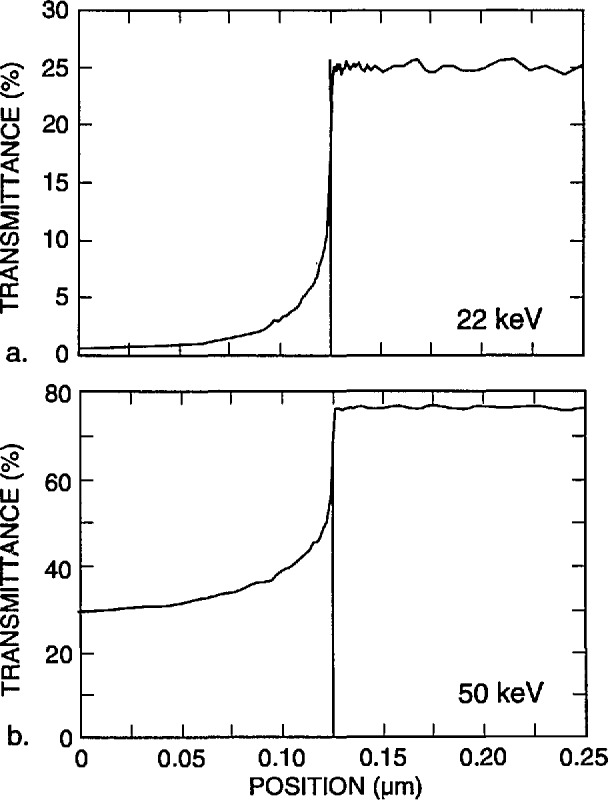
Monte Carlo modeling of the electron fraction transmitted through the x-ray mask, (a) 22 keV electron beam, (b) 50 keV electron beam.

Figure 7 shows the fraction of the electrons that are expected to be transmitted and collected by a detector with a diameter of 12.5 mm located 5 mm from the lower surface of the membrane, with a diode cutoff energy of 4 keV, as a function of the position of the incident beam. No energy filter was included in this calculation. The energy of the incident electrons is 22 keV in Fig. 7a and 50 keV in Fig. 7b (10 000 trajectories were simulated for each point). As demonstrated in these diagrams, the contrast at the edge is better for the lower accelerating voltage electrons in spite of the fact that only about 10% of the electrons are collected by the detector, compared to over 90% for 50 keV. The reason is that practically no electrons are transmitted when the beam is incident on the center part of the strip for an incident energy of 22 keV, as opposed to about 30% collected electrons passing through the absorber at 50 keV. The change in the number of transmitted electrons as the beam passes over the edge is quite sharp, leading to a precise value of the width (as discussed later). Modeling also can provide the fraction of the maximum number of transmitted electrons that corresponds to the edge of the strip. It is apparent that a trade-off between contrast and resolution is necessary. By the addition of the copper filter we were able to increase the accelerating voltage to improve instrument resolution, but at the same time maintain high contrast. For the metrology work a beam diameter of 10 nm has been used and an accelerating voltage of 30 kV with a copper foil filter having a cut-off at about 16 keV. The 16 keV cut-off was determined experimentally as shown in Fig. 4b.

#### 2.5.1 Detector Placement

In many typical transmission electron applications in which high-energy electrons are aimed at a target with the purpose of detecting cellular ultrastructure (or other types of samples), the optimum type of detector is one placed at a relatively long distance from the sample. Such a detector covers a small solid angle and collects electrons that have gone through the material essentially undeflected through a very thin sample. Contrast of the ultrastructure is enhanced by insertion of an aperture preceding the detector. This would be the typical philosophy adopted by those commonly working with a scanning transmission electron microscope (STEM), where the entrance angle to the electron detector is typically about 1 × 10^−4^ rad. The overall construction of the STEM instrument permits more flexibility in the detector and aperture placement than the SEM, although often at the expense of a limited sample size. This limited-solid-angle approach to the detector was the initial philosophy adopted in the early x-ray mask metrology study based on the typical TSEM geometry [5,6]. In that work, the electron detector had an. entrance angle of about 1.7 × 10^−2^ rad. This resulted in a poor signal-to-noise ratio and thus limited the ability to achieve small spot diameters. This also resulted in a very limited field of view as well as excessive noise amplification (60 Hz) in the signal.

X-ray mask metrology presents a different situation from that described above. A very small fraction of the electrons, only a few in 10 000, satisfy the conditions that allow them to be collected by a small-angle detector unless the beam energy is much higher than 50 keV. As shown in Fig. 6, the electrons in the modeled energy range suffer multiple scattering events. Those that emerge from the lower surface of the membrane may or may not be collected by the detector. A detector with a large acceptance angle, located close to the lower surface will collect a significant fraction of the transmitted electrons. The edge region is characterized by a rapid change in the total number of transmitted electrons as the beam passes over the edge of the gold strip, as shown in Fig. 7.

As the incident energy decreases from 50 keV, the ratio of the two on-feature and off-feature current levels in Fig. 7 increases until it reaches a maximum in the neighborhood of 22 keV (unfiltered), and then sharply decreases again as the number of electrons transmitted through the membrane alone becomes very small (i.e., the signal decreases to zero) near 20 keV. Therefore, for this particular metrological application which differs from the customary STEM or TSEM application, it is favorable to collect as large a fraction of the transmitted electrons as possible. Consequently, a large, wide-angle detector was located close to the sample (as described below). Within the practical limitations due to the configuration of the scanning electron microscope, this strategy leads to stronger signal and good images of the structure(s) of interest. The excellent signal-to-noise ratio demonstrated by this configuration permits minimum spot diameters and thus, potentially higher resolution of the edge and lower edge uncertainty than with the original commercial configuration used for this purpose. It is also possible to obtain solid-state diodes such as the one currently under test as large as 12.5 mm in diameter. Thus, much of the x-ray mask pattern can be viewed directly even at low magnifications. However, it should be noted that diode uniformity and bandwidth limitations require further investigation. Alternatively multiple diode detectors, microchannel-plates or scintillator detectors could be used. However, due to our special space limitations, the diode detector proved to be the most appropriate.

Early work [4] demonstrated an experimental comparison between the two types of detector configurations. The difference between the conventional detector configuration and the one where there is a wide solid angle of acceptance and placement is about 5 mm from the sample was shown to be significant. There was increased contrast without any degradation of edge definition in the close detector arrangement. In fact, in this mode of electron collection, the signal was so great with the commonly used primary electron-beam currents for this type of work, that the gain of the amplifier required substantial reduction to avoid saturation effects. Under these conditions the spot diameter of the SEM could be reduced to the minimum possible, still with acceptable contrast. These results were first predicted by the modeling work and subsequently confirmed experimentally. This is the type of detector used throughout this work.

### 2.6 Specimen Charging

Specimen charging is always a concern in scanning electron microscopy. The x-ray mask used in this study has the chrome plating base left in place. This provides a grounded conductive layer under the isolated structures and over the insulating layers (Fig. 1). Unfortunately, the chromium coating can have structure. This structure adds to the “noise” in the measurement scan (discussed later).

The surface of the mask was also carefully grounded by using a grounding clip. The potential effects of specimen charging were also tested by partially coating a test mask and comparing with the data. For this work it was determined that where this specific all-conductive mask construction was concerned, specimen charging was not a major concern. For the development of standards, often it is necessary to optimize the sample for the metrology. X-ray masks constructed without insulating layers might circumvent charging problems if suitably grounded. However, no masks without the chromium layer were tested. X-ray masks with insulating membranes (e.g., silicon nitride) could have greater charging problems as well as those masks that have another insulating layer above the support membrane and absorber.

## 3. Results

### 3.1 Electron Beam Interaction Modeling

**Fig. 8:**
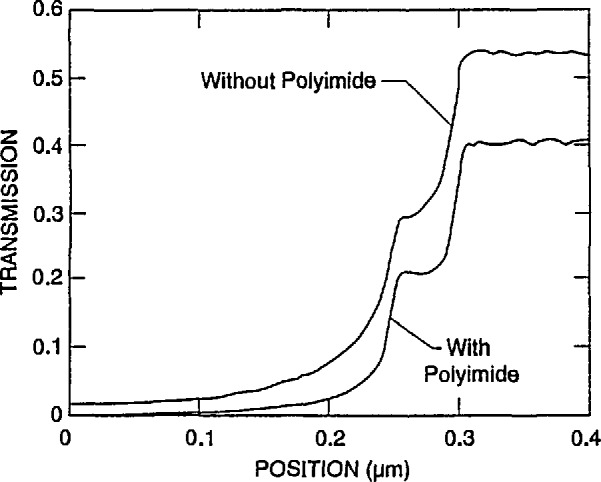
Comparison of the modeled transmitted electron line profile with a 4° wall slope with (dash) and without (solid) the polyimide layer.

There are currently no standardized x-ray mask fabrication techniques. This area is currently undergoing evaluation and experimentation regarding the most effective construction of the masks. This evolution process necessitated the development of reasonably flexible computer modeling code. Changes in the mask structure during this work necessitated that the modeling be run with the polyimide layer in place for early test masks, as well as removed (actually with this layer still included, but with zero thickness) for the later ones. The results, as expected were similar, but varied from the original by an absolute increase in the level of electron transmission of about 14% in the membrane region and 2–3% in the absorber region (Fig. 8). The fundamental characteristics of the transmission profiles did not change significantly within the “noise” of the modeling. Therefore, the results described here pertain to either mask design (unless specifically indicated). The modeling parameters used for Fig. 8 were those described previously for metrology as well as a slight wall slope (as described below).

**Fig. 9:**
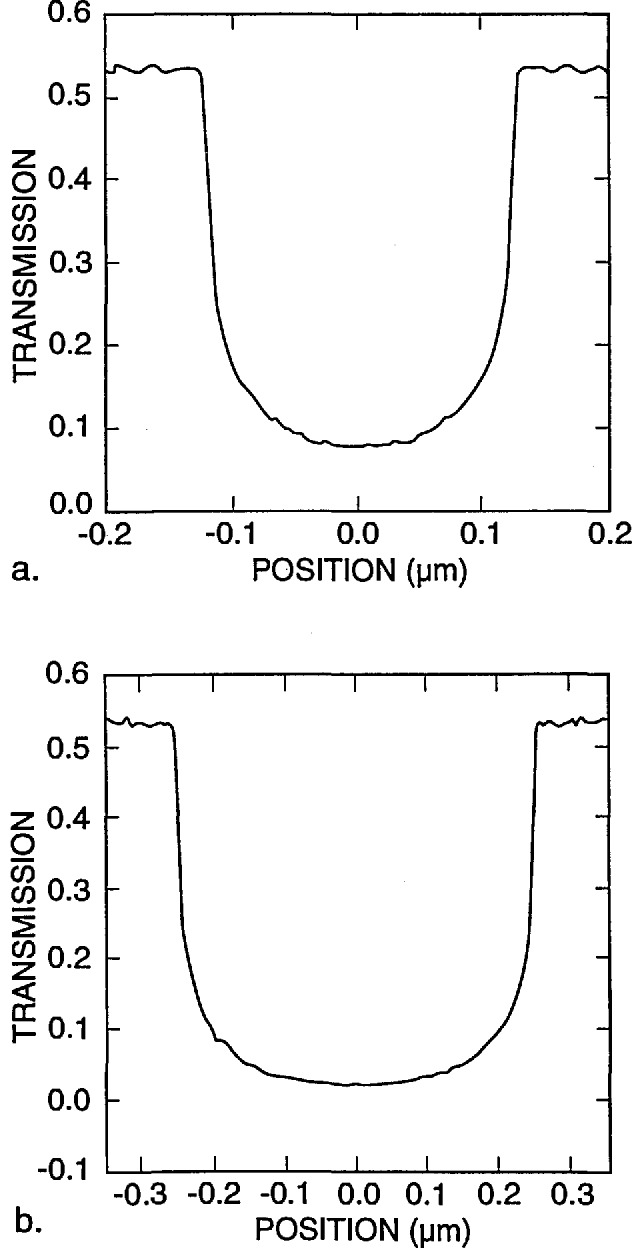
Monte Carlo image profile of absorber lines with perfectly vertical walls, (a) 0.25 micrometer line and (b) 0.5 micrometer line both computed with 0° slope to the wall.

**Fig. 10:**
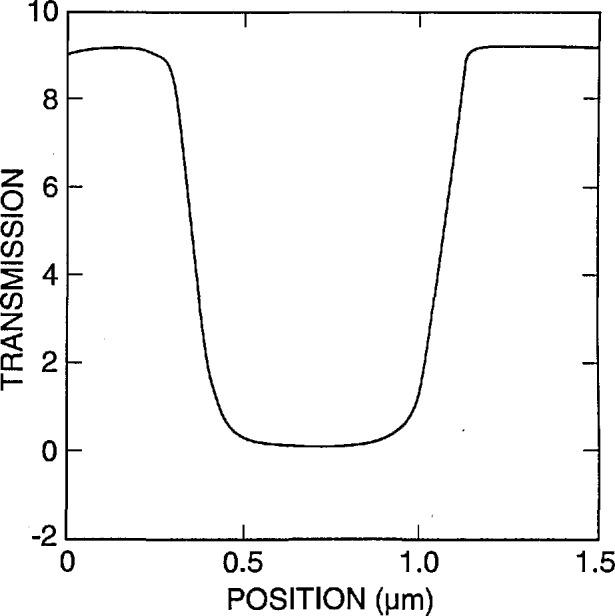
Experimental data obtained from the NIST metrology instrument of a 0.5 μm nominal line obtained by using the methods described in the text.

Figure 9 shows the modeling results for a 0.25 and a 0.5 μm line with vertical walls. If a perfect vertical wall structure could be manufactured, the determination of the edge would be quite straightforward. Unfortunately due to the fact that we are dealing with “real world” structures and not the ideal structures assumed in computer models, it is not so straightforward. Figure 10 is a plot of the experimental data obtained from the metrology SEM from a 0.5 μm line. It can be seen from comparison of Figs. 9 and 10 that the experimental data obtained are broader than the computed profile for a vertical wall. A substantial amount of effort was expended in order to determine the major factors causing variations in the line profile from the computed values. Some of the contributing factors found to cause a deviation from the ideal are discussed in the following sections.

#### 3.1.1 Wall Verticality

**Fig. 11:**
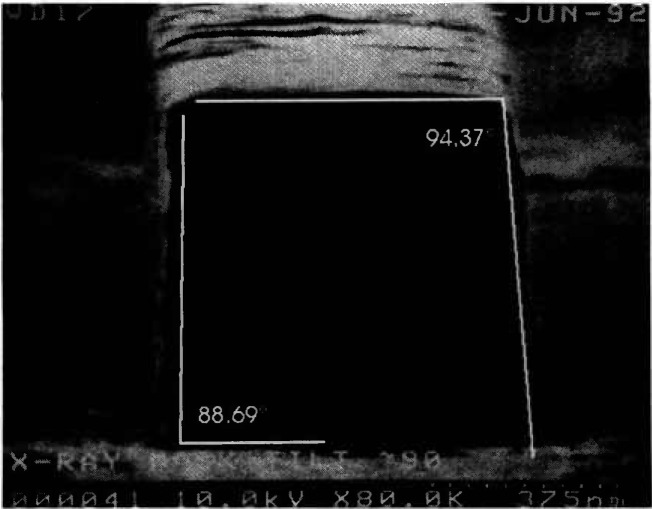
Scanning electron micrograph that has been scanned into the Isaac image analysis system demonstrating the approximate 3° slope to the sidewalls.

With the best conditions and stage capabilities possible in the NIST field-emission SEM, it was determined that the wall slope of a line on a broken piece of the x-ray membrane is about 2°–3° (Fig. 11). Because of fundamental FESEM stage limitations, only broken pieces of the mask could be viewed at high tilt. The numbers are obtained from non-cross sectioned samples since to cross section an x-ray mask would require the development of precise metallurgical-like mounting and polishing techniques (which was not part of this study). Therefore, since compound angles are involved in the micrographs, these angles are not exact but are assumed to be reasonable.

**Fig. 12:**
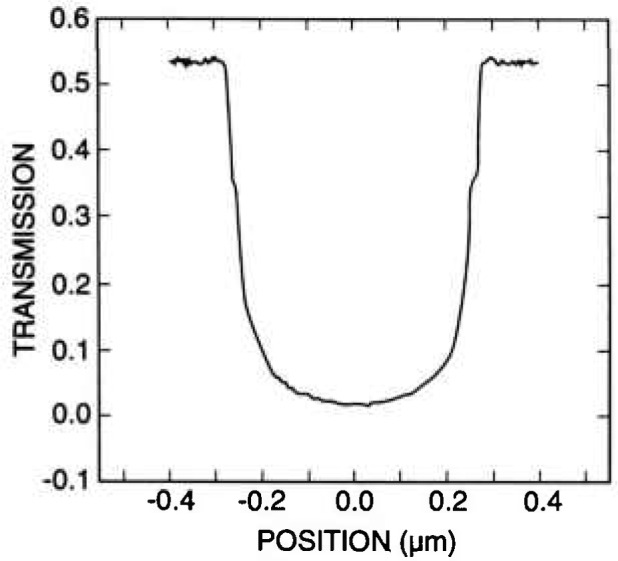
Monte Carlo image profile of a 0.5 μm gold line with a 2° wall slope.

The TSEM method is very sensitive to wall slope. Small angular deviations from vertical result in significant changes in the profile. Monte Carlo modeling of a 2° sloped-absorber wall (Fig. 12) begins to approach the experimental data, as shown, in Fig. 10.

**Fig. 13:**
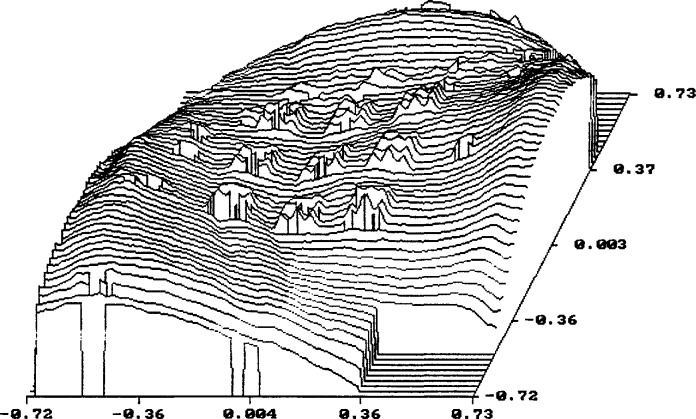
WYKO 6000 Phase measuring interferometer flatness measurement of the x-ray mask surface demonstrating a 0.5 μm peak-to-valley variation across the surface of the mask (courtesy of Mr. Chris Evans).

Wall verticality issues are not only associated with structure fidelity but also surface flatness. Surface flatness measurements made with a WYKO 6000 Phase Measuring Interferometer [30] on several of the test masks, have shown that there is on average a 0.5 μm peak-to-valley variation across the surface of the mask within the measured window (Fig. 13). This variation could also contribute to the inaccuracy of the measured result. Local waviness across the x-ray mask can result in variations in local surface tilt which can then be translated to differences in the resulting video profile and eventually the measurement.

**Fig. 14:**
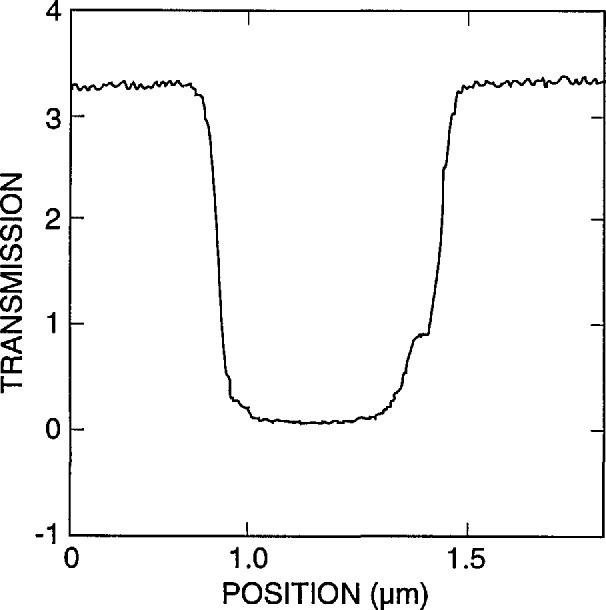
NIST metrology instrument measurement scan of an absorber line which has had 4° of experimentally induced angle into the position of the mask. Note the prominence of the notch on the right side of the graph. If the induced angle is placed 90° from the first measurement the notch appears on the opposite side. Also notice that the verticality of the opposite side of the scan approaches the modeled data for the 0° case since the composite wall angle has been compensated by the shimming of the mask.

A third factor associated with wall verticality is that of positioning within the measurement instrument. The surface of the mask must be perfectly perpendicular to the electron beam. If the SEM sample stage itself were to demonstrate a 2° tilt from normal to the beam this is added to the 2° already present on the structure. This effectively results in a vertical wall on one edge and a 4° slope on the other edge. This effect has been able to be demonstrated experimentally as shown in Fig. 14. This concept is essentially equivalent to tool induced shift (TIS) found in other forms of measurement equipment. The effect was verified by reversing the application of the angle by 180° as done in standard TIS testing. This stage wobble factor underscores the need for extremely precise SEM stage construction in inspection instruments with this technique. A stage accommodating a 12.7 cm (5 in) x-ray mask may need to travel up to 25.4 cm (10 in) to view and measure all the structures necessary. Such a stage must be aligned perpendicular to the electron beam well within 1° for the entire travel. The stage must also demonstrate roll during travel of less than 1° as seen by the sample as it travels or the roll will translate into tilt on the sample. Measurements using the secondary or backscattered electron techniques will also be affected, but the magnitude of the effect was not a part of this study.

#### 3.1.2 Wall Edge Roughness

**Fig. 15:**
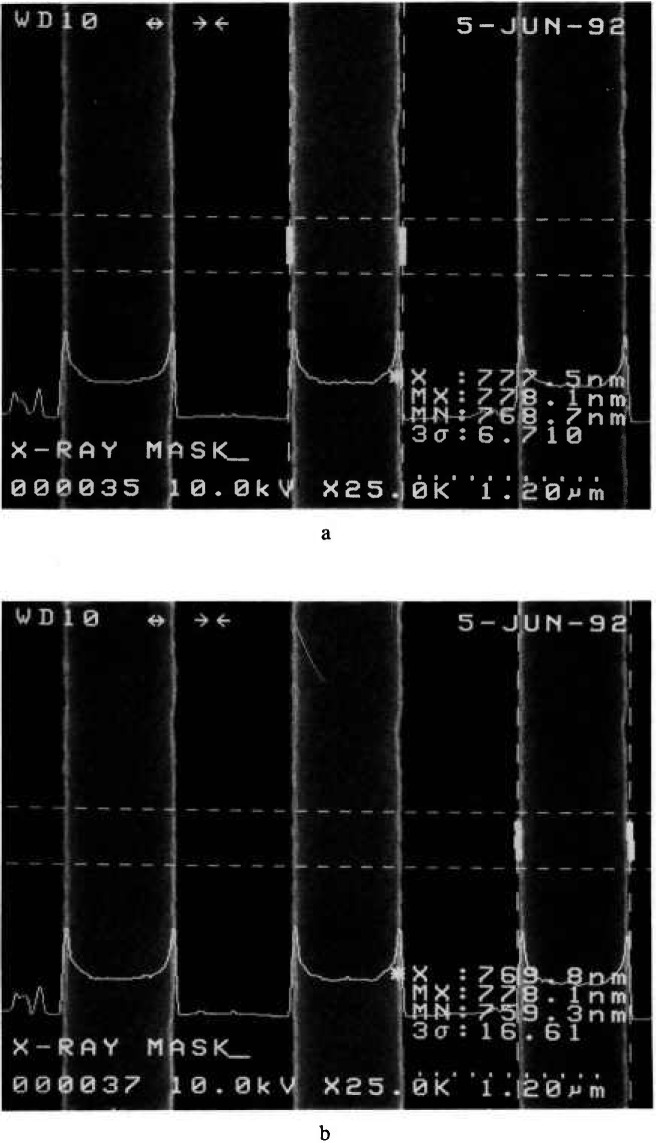
Scanning electron micrographs and beam scanned measurements of the gold absorber lines demonstrating the edge precision of the lines, (a) Position 1 and (b) Position 2. Note that depending upon the position on the absorber line where the measurement is taken, the 3*σ* value for the precision varies from 6.7–16.6 nm.

There is about a 7–16 nm edge wall roughness that can be attributed to the structure or graininess of the gold absorber and other factors as seen in Fig. 2 and measured by the digital beam scan measurement in Fig. 15. This surface roughness randomly affects the effective slope of the edge as seen by the electrons and can increase the apparent deviation from vertical by about 2° from its estimated geometrical value. This makes the effective wall slope, as seen by the electrons in the NIST metrology SEM, to be about 4° (explained later).

#### 3.1.3 Edge Rounding

The edge of the absorber is rounded and not sharply delimited as shown in Fig. 2. This affects the transmission profile in those regions where these rounded edges are interacting with the electron beam (e.g., when the electron beam is incident on the top of the line near the edge). Edge rounding is not serious *vis-a-vis* determining the edge location in the present measurements. However, it should be included in future versions of the Monte Carlo modeling.

#### 3.1.4 Wall Foot

**Fig. 16:**
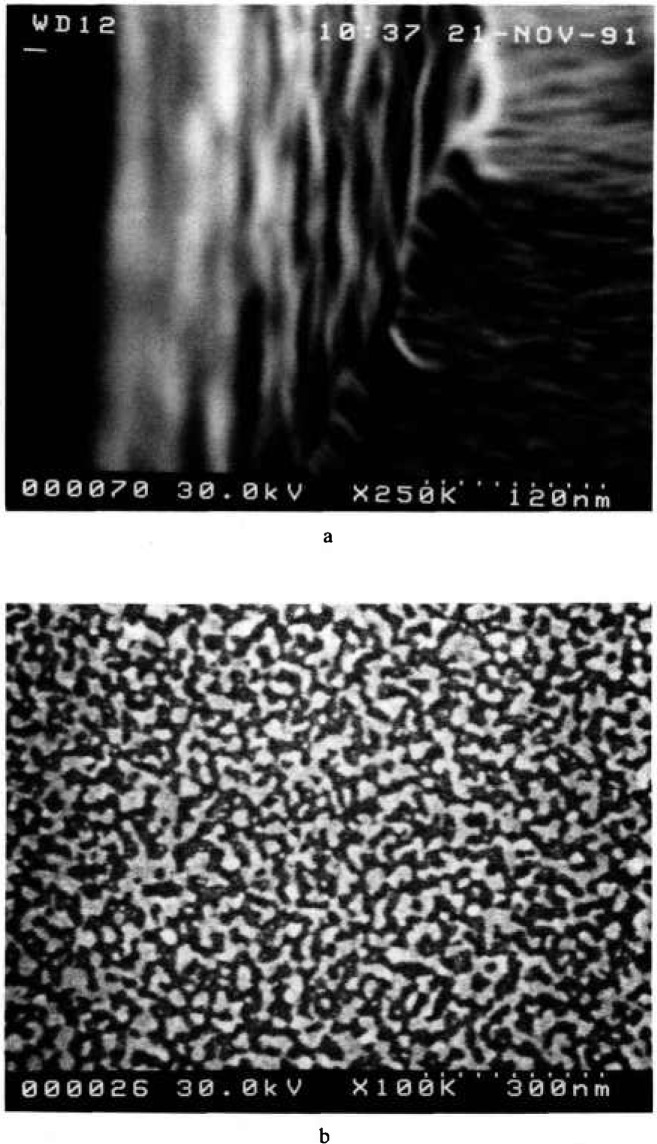
Scanning electron micrographs of the x-ray mask structure, (a) Wall foot showing the material at the base, (b) Structure of the chromium.

Many of the “real-world” structures have a foot associated at the base which can be as large as 30 nm in size (Fig. 16a). The effects of this foot to the measurement are similar to edge rounding and is most evident when the electron beam is incident on or near the foot. The material composition of this foot is not known but it is presumed to be material left over from previous processing steps. Like edge rounding, it appears not to be a serious limitation for the present measurements. This structure (depending upon its composition) could become a problem especially if truly vertical walls were fabricated.

#### 3.1.5 Chromium Structure

The chromium can have a structure as evidenced by high resolution SEM imaging (Fig. 16b). This structure adds noise to the measurement but seems to have little effect on the metrology.

#### 3.1.6 Contamination

**Fig. 17:**
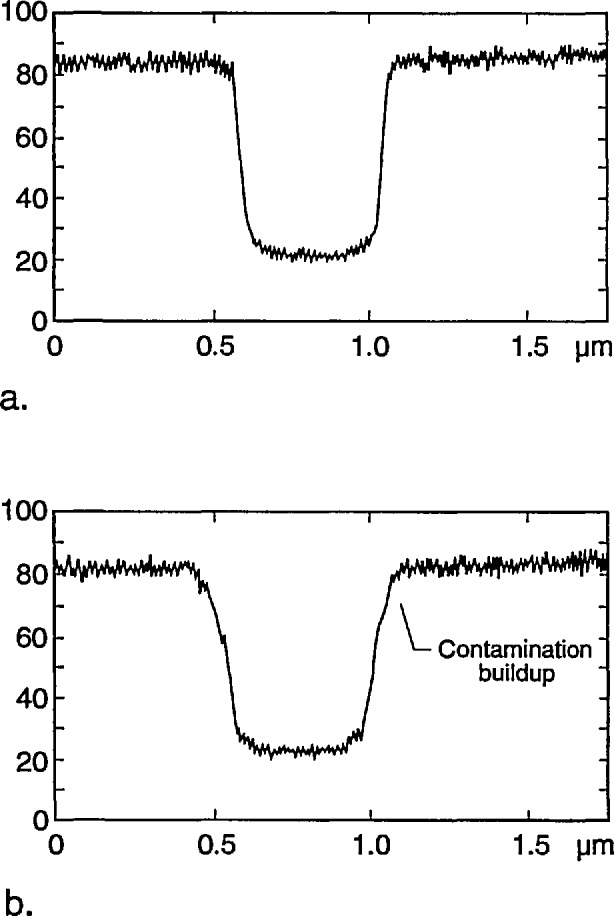
Effects of sample contamination on the measured profile, (a) Original scan, (b) Scan after intentionally allowing the sample to become contaminated.

Hydrocarbon contamination of the sample deposited by the electron beam measurement can contribute to the measurement imprecision in two ways. First the buildup of the contamination appears initially at the base of the absorber structure, therefore broadening the base of the measurement profile (Fig. 17). Second, contamination build-up can tend to attenuate the collected transmitted electron signal and thus alter the actual measurement threshold. The figures shown were intentionally contaminated for an extended period of time. Under normal measurement conditions this should not be a problem.

### 3.2 Analysis

**Fig. 18:**
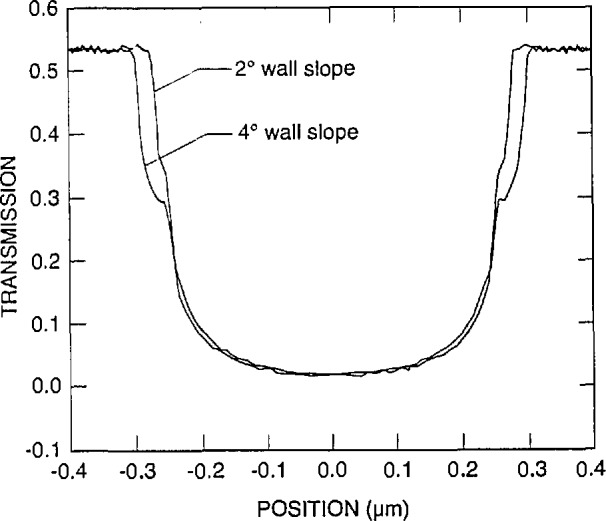
Monte Carlo modeled data for 0.5 μm gold line with a 2° wall slope and a 4° wall slope superimposed.

The six factors described above, with the first two (wall verticality and edge roughness) being the dominant ones, contribute to the differences seen between the observed transmission line profile obtained with the TSEM technique on “real world” samples and the Monte Carlo modeling result for an ideal line under the same conditions of measurement. It was therefore necessary to model a line for each case with the measured geometrical slope of 2°, as well as one with an effective slope of 4° for the various cases. Figure 18 shows the data for the 0.5 micrometer line with the two wall slopes superimposed for each case.

It has been shown that the edge of the absorber line is not a perfect vertical wall. Instead there is a widening at the bottom which results from its trapezoidal shape as well as rounding and roughness. The computer modeling code, for this case, is based on a simple trapezoidal line with known height, measured to be 0.7 μm, and variable wall angle. The measured wall angle was about 2°, but a best fit to the measured lines was with an angle of 4°. The extra angle is a way to include the effects of roughness and rounding, which make the variation of transmission more gradual. Thus, there is an ambiguity in interpreting the predicted position of the edge. One can either make a best fit with the measured 2° slope and take the position of the edge from it or one can use the better fit with the 4° slope and take the edge from it. The former method attributes disagreement in fit to the nonideality of the trapezoidal line and then assumes that the edge is still properly determined by neglecting these effects. The latter method attempts to incorporate these effects in the model and extract an edge position that in some sense includes them. Both approaches have been used and the results from the two methods are expected to bracket the true edge position.

**Fig. 19:**
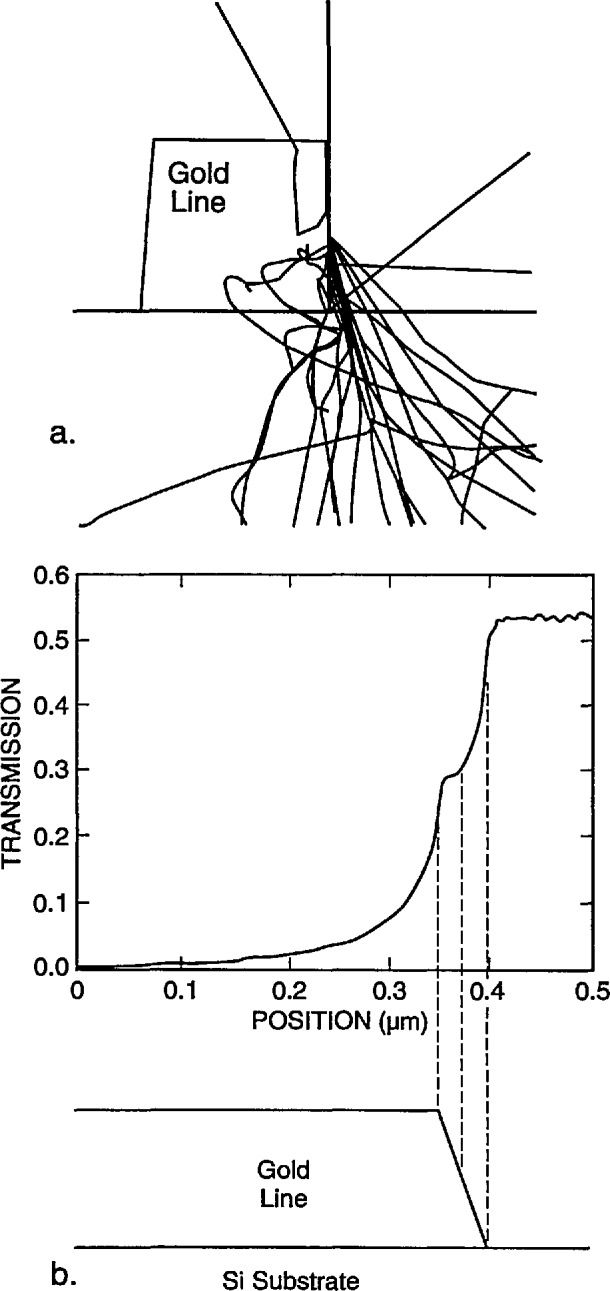
The effects of the electron scatter from a sloped edge of the absorber, (a) Trajectory plot showing the reflection of electrons from a 4° sloped surface, (b) Diagram showing the reflection of electrons from the edge as related to the structure and modeled profile.

**Fig. 20:**
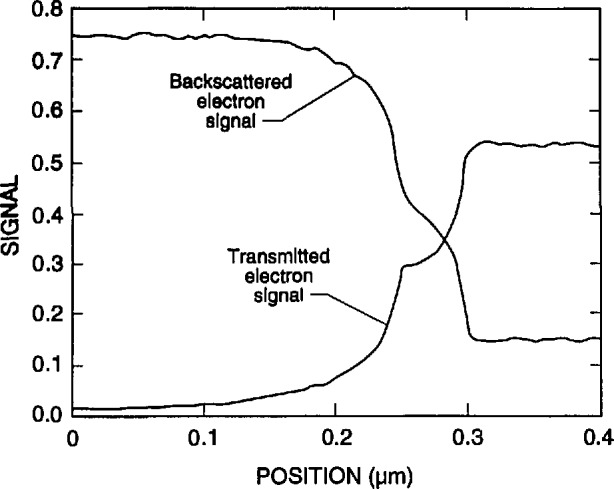
Monte Carlo modeled data of the transmitted electron signal and the backscattered electron signal showing the appearance of the characteristic notch in both modes of electron detection.

A very interesting phenomenon was observed in the simulation of the transmission across the edge of an absorbing gold line on a silicon membrane. When the beam lies wholly on the sloping edge of the trapezoidal line, the electron transmission remains relatively constant especially along the upper half of the slope. The signal constancy is enhanced by the removal of lower energy electrons by the copper detector filter. This effect occurs because the electrons that strike a steeply sloping edge can escape easily from the surface and continue down to the detector. In a sense, the electrons are reflecting off the surface. The point of contact along the face is then not very important because the phenomenon is nearly the same at all points. Figure 19a is a trajectory plot of 20 electron trajectories showing how the electrons exhibit this behavior. The height and width of the modeled line are both 0.7 μm, and the beam intersects the middle of the 4° trapezoidal face. The beam diameter is 10 nm. Many of the electrons exit from the face to form the stream that is then collected by the detector. This phenomenon can be used to determine the position of the edge if it can be seen in the measured TED mode image. If the necessary spatial resolution can be achieved, then this signature can be observed provided that the surface is not so rough that it is completely blurred. The effect of this phenomenon on the modeled electron transmission, as compared to the structure of Fig. 19a, is shown in Fig. 19b. This figure shows the right-hand edge of the line. The line center is taken as the left-hand edge in the figure. The transmission becomes relatively constant when the beam is wholly on the face and not much below the center of the face. Further modeling of the edge roundedness and the effect of the foot at the base would help to understand better the transition points before and after this notched region. The same effect occurs in the backscattered electron signal and it may also become useful in finding the edge in this method of electron collection as well (Fig. 20) but has yet to be confirmed experimentally. This phenomenon is currently being explored further.

**Fig. 21:**
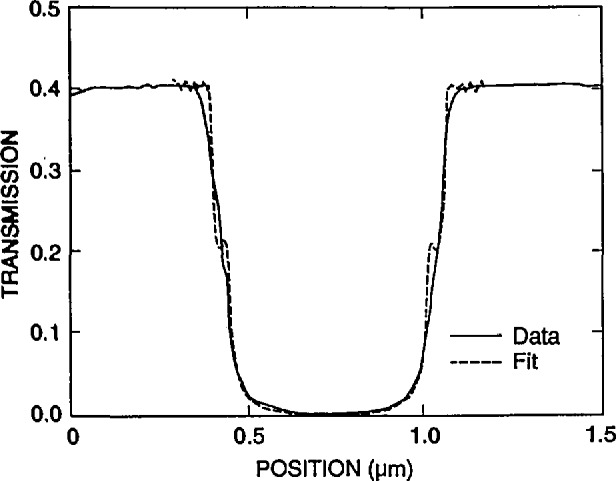
Comparison of the Monte Carlo modeled data for a 4° wall slope to the actual fitted experimental data.

As the wall angle increases from vertical to 4° (and beyond), this characteristic notch begins to appear as a ledge in the model profiles. This is an important observation because the model tells us that this ledge occurs when the electron beam is incident on the sloping edge of the assumed trapezoidal line. This is confirmed in the experimental results shown in Fig. 14 where the effective vertical wall of the right side with no notch contrasts with the 4° slope of the left side which has a prominent notch. Just as in the model, as the wall slope decreases, the position of this notch moves up the profile to ultimately become obscured at verticality. If the actual laser interferometer/SEM data is fit to the 4° modeled data, excellent agreement is obtained (Fig. 21)—except in the region of the actual notch. Perfect agreement of the experimental data obtained from the NIST instrument to the modeled data, in this region, is difficult for two reasons: 1) the current NIST instrument has a flat final lens and is operating at a relatively long (12 mm) working distance to accommodate the laser interferometer stage assembly. Under these conditions the instrument operates at about 10-nm (or poorer) ultimate resolution previously shown in Fig. 3. Unfortunately, the size of the notch is approximately 8–10 nm within the 4° slope range (less for 2°) and thus is not always resolved; and 2) the factors associated with surface roughness and rounding of the edge are random and the notch may appear on one edge of the profile but not on the other and can be blurred. Then the question becomes: is the character found on the measured profile real or is it noise? Indeed, the characteristics of the notch are within the resolution capabilities of a good field-emission SEM. The notch then can sometimes be unambiguously detected by its consistency from scan to scan whereas the noise is not consistent (see continued discussion on this point below).

### 3.3 Measurement Criteria

**Fig. 22:**
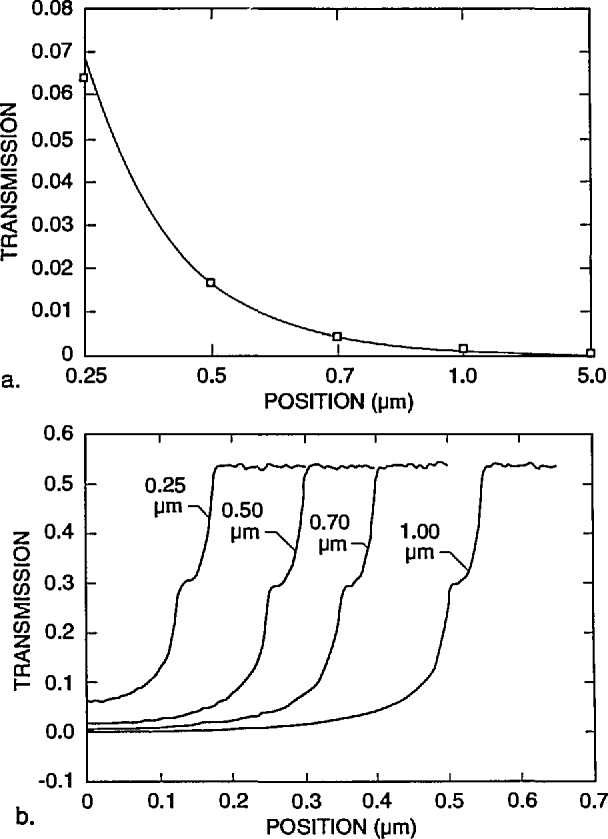
Effect of electron transmission on the location of the edge, (a) Relationship between the percent minimum transmission as related to the size of the absorber structure as modeled, (b) Monte Carlo modeled plots of the threshold variation as a function of structure size for 0.25, 0.5, 0.7 and 1.0 μm width lines with a 4° sloped wall. Both the 0.35 and 0.75 μm thresholds have been interpolated from these data.

**Fig. 23:**
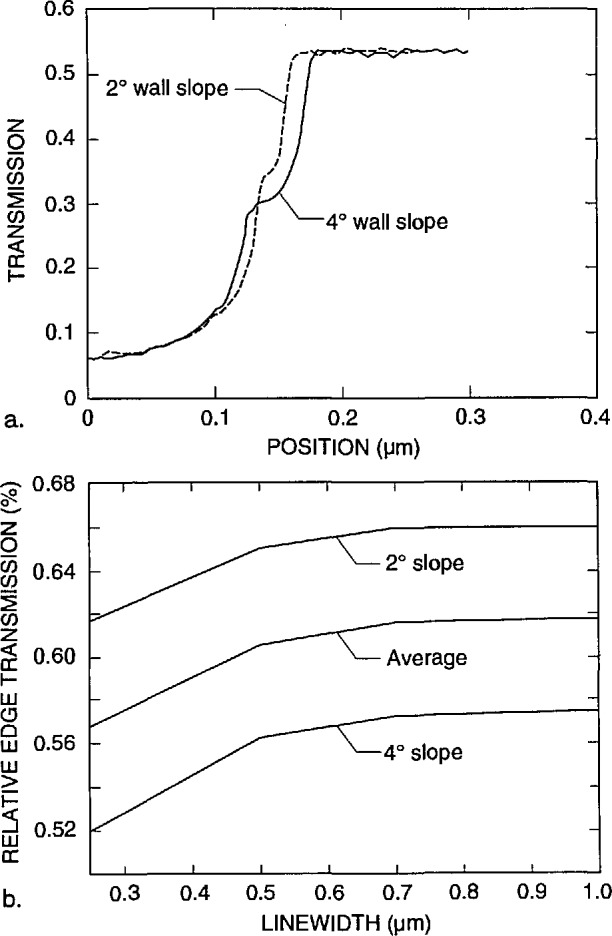
Relative edge transmission, (a) Plot of the relative edge transmission of a 0.25 μm line as related to the nominal linewidth for the 2° wall case (dashed line) and the 4° wall case (solid line), (b) Plot of the calculated relative edge transmission of various width lines from 0.25 to 1.0 μm and the average between the two where the measurements were made.

For the purposes of the present study, it will be assumed that the effective linewidth for x-ray masking purposes is the width at a point 50% down the slope of the absorber line. This assumption could be refined in the future by comparison of linewidths on x-ray masks measured by the present technique and linewidths actually produced by using the measured masks. Fortunately, 50% down a line is geometrically the same on a 2° or 4° edge slope. However, the modeling reveals that the notch position on the video profile (and thus the position of the edge on the profile) is a function of **both** edge slope **and** linewidth for linewidths less than about 2.0 μm. This is due to the fact that as the linewidth decreases below about 2.0 μm, the minimum transmission increases because of leakage of electrons out from both edges. This means that as the linewidth decreases, more transmission occurs and the baseline level of the profile increases (Fig. 22a). Even an infinitely large gold line will have some base level of transmission (0.002% of incident electrons) and as the line decreases in size this will rapidly increase as shown in Fig. 22b. This means that there would also be a vertical shift in position of the “edge” location as interpreted from the model. Furthermore, the absolute transmission at the edge depends on edge slope for any linewidth because the amount of leakage out the edge depends on the slope (Fig. 23).

The criteria for determining the edge position, for the masks used in this study are summarized in Table 1. These are determined from the modeling results as follows: the 50.0% point of the face of the slope is located at the 52.0% transmission point on the TSEM profile of a 0.25 μm line with a 4° effective slope and at the 61.7% point for a 2° sloped line. Since we can argue for both 2° (real) slopes and for 4° (effective) slopes, we average these values to obtain an “edge” criterion at the 56.9% point. For a 0.5 μm line with a 4° effective slope the position is at the 56.2% point while for a 2° slope the point is at 65.0% (Fig. 23a). The average is 60.6% and is where the measurement is made (Fig. 23b).

The resulting difference in linewidth between the *2°* slope percentages and 4° slope percentages is accepted as a systematic unknown component of inaccuracy. This difference is estimated to be less than 10 nm for each linewidth measurement for edges defined at 50% down the absorber line and does not contribute to errors in the pitch measurements since it is a self-compensating measurement. This component of inaccuracy would decrease for smoother walls and/or smaller edge slopes.

#### 3.3.1 Measurements

**Fig. 24:**
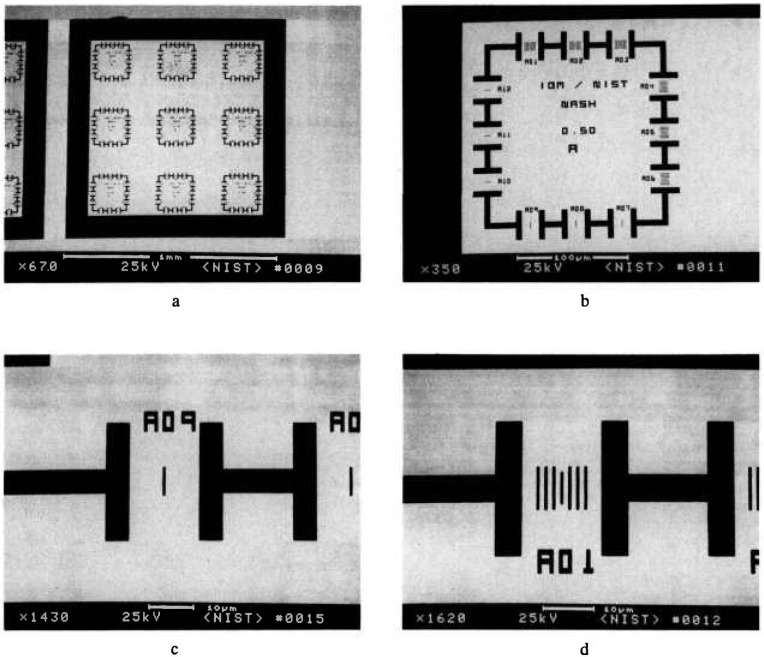
Measurement data. Micrographs of a representative x-ray mask feature having a nominal 0.5 μm absorber structure measured in the NIST metrology SEM. (a) Low magnification image showing absorber pattern array, (b) Individual pattern with nominal 0.5 μm absorber structures, (c) Isolated absorber line, (d) Pitch array of the absorber lines.

**Fig. 25:**
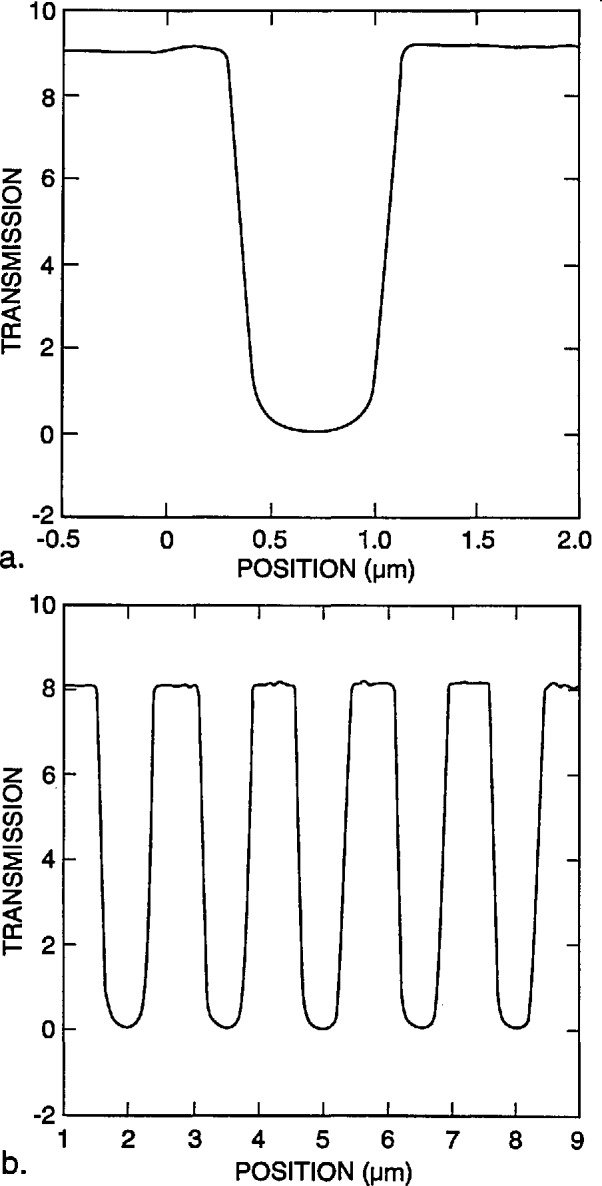
Measurement data. Actual laser stage data, using the TED mode, from the NIST metrology instrument of the 0.5 μm absorber structures, (a) Isolated line used for width measurement, (b) Array of lines used for both width and pitch measurements.

**Fig. 26:**
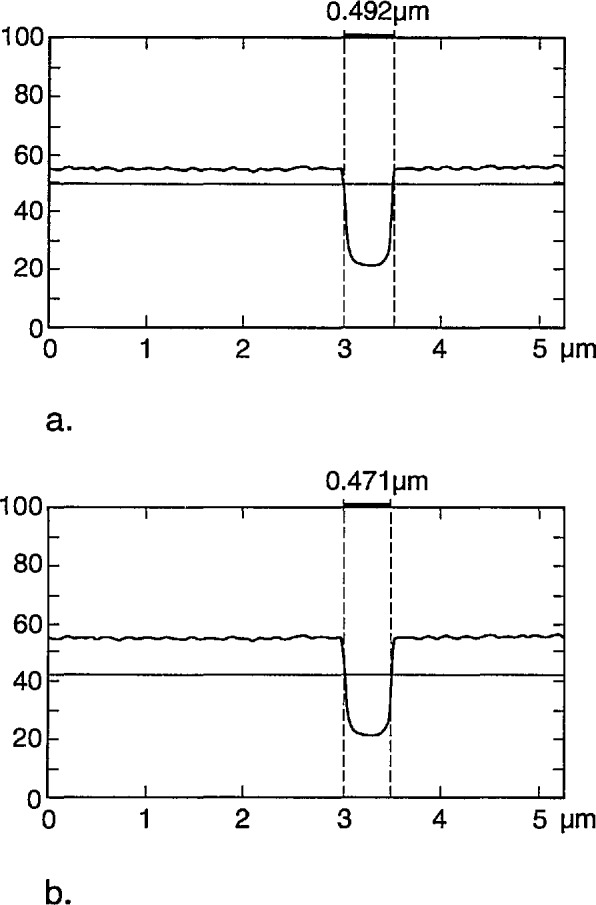
Beam scanned comparison measurement using the classic AMRAY A-90 measurement system installed on the NIST metrology SEM: (a) 20% negative autothreshold setting and (b) 40% negative autothreshold setting.

A series of x-ray masks were measured by using the metrology SEM and the criteria, as described above for the edge location. Example measurements are shown in Table 2. Examples of representative 0.5 μm lines for pitch and width measurements are shown in micrographs of Fig. 24 a–d and the profiles of these lines are shown in Fig. 25 a and b. The pitch measurements of these lines represent about 25 000 original data points per measurement and the width measurements represent about 8000 original data points per measurement handled in the manner described previously. The above measurements compare favorably with the beam scanned data from the same measurement instrument for a 0.5 μm line depending upon the threshold setting chosen (Fig. 26).

The measurement results, to date, are quite encouraging and indicate that the present technique has resulted in an estimated edge-location uncertainty as low as 10 nm with the modeling. This performance could be further improved by improvement in the wall edge verticality and the surface roughness. Increases in the number of pixel points available for the measurement, in the use of high-brightness, high-resolution field-emission electron optics, or the development of measurement algorithms based on the electron-beam modeling would improve the data handling but would not improve the uncertainty of this measurement. This occurs because for the level of accuracy required, the physical limitation imposed by the measurement subject is the limiting factor.

### 3.4 “To Notch or Not to Notch”

**Fig. 27:**
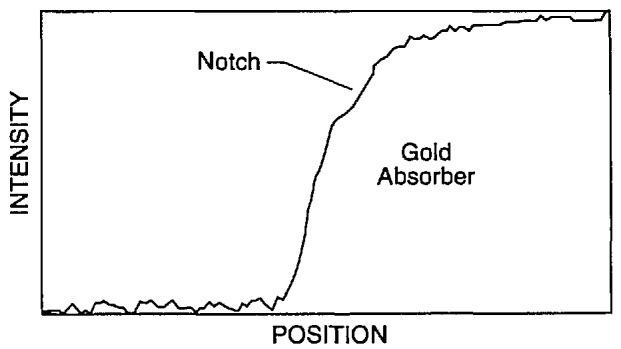
Digitized field emission scanning electron micrograph demonstrating the presence of the characteristic notch on one side of the profile. Note that the data have been inverted.

**Fig. 28:**
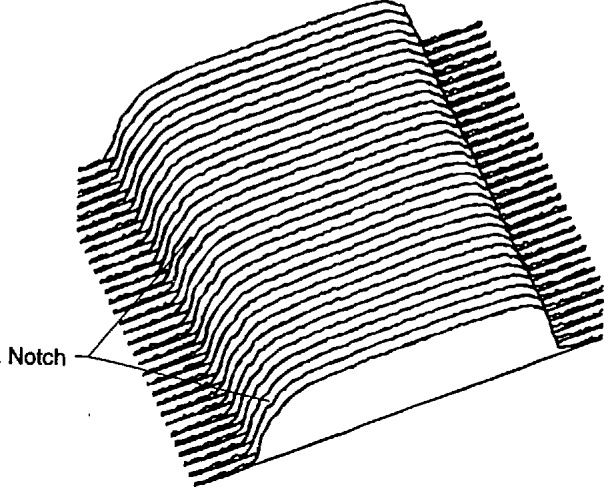
Image analysis of the digital field emission scanning electron micrograph demonstrating multiple line scans down a gold line showing the presence or absence of the characteristic notch along the left edge depending on the surface roughness. Note that the data have been inverted.

The question “can the notch be resolved?” was also studied. The complete “test” mask on the TED assembly was carefully installed in the NIST FESEM and the characteristics of the image of the transmitted electrons studied by using the standard beam scanning mode. This image was then transferred and stored in the Isaac image analysis system. When the resolution and signal-to-noise level are adequate (as they are in the FESEM) a notch was resolved (Figs. 27 and 28).

Figure 28 shows that the resolution of the notch structure is sometimes only on one edge due to local grain interference, rounding or roughness, which complicates matters somewhat and can be seen to vary as the beam is scanned across the line. This characteristic is good for precise metrology if this notch can be precisely imaged in the measurement instrumentation and algorithms developed to exploit this characteristic. We are unable to image this notch routinely (as discussed previously), in the metrology SEM, but our beam-scanned measurements with the FESEM demonstrate its location in the profile (Figs. 27 and 28).

### 3.5 Imaging and Particle Detection

**Fig. 29:**
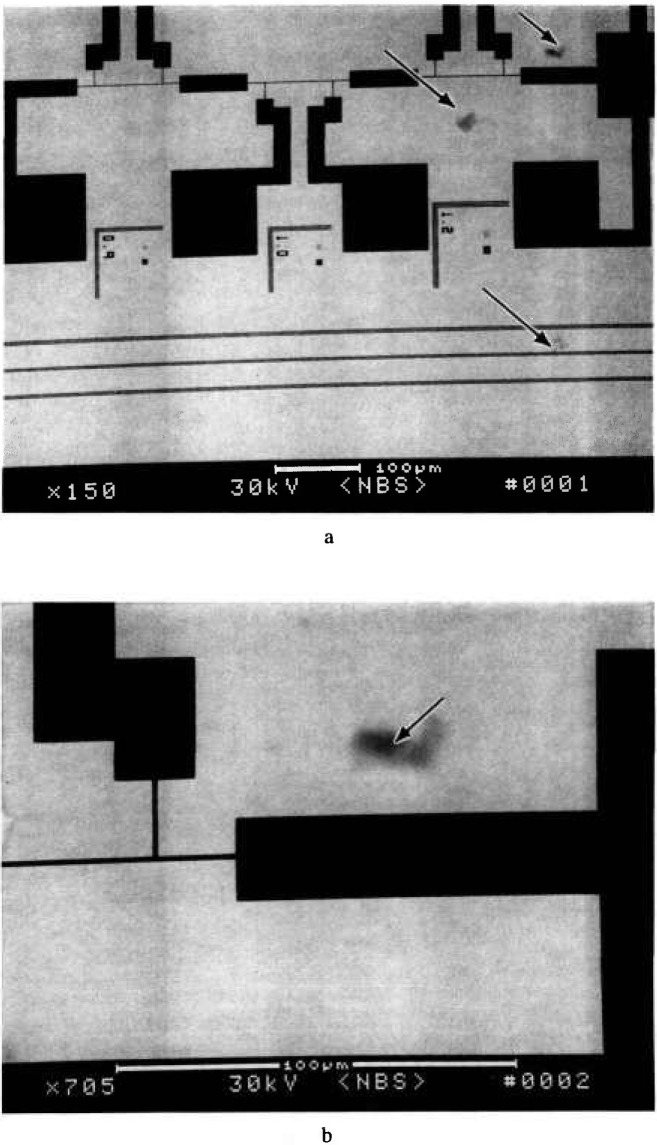
Particle detection using transmitted electron detection, (a) Low magnification micrograph demonstrating a test pattern with several particulate defects, (b) Higher magnification of the area with the defect.

**Fig. 30:**
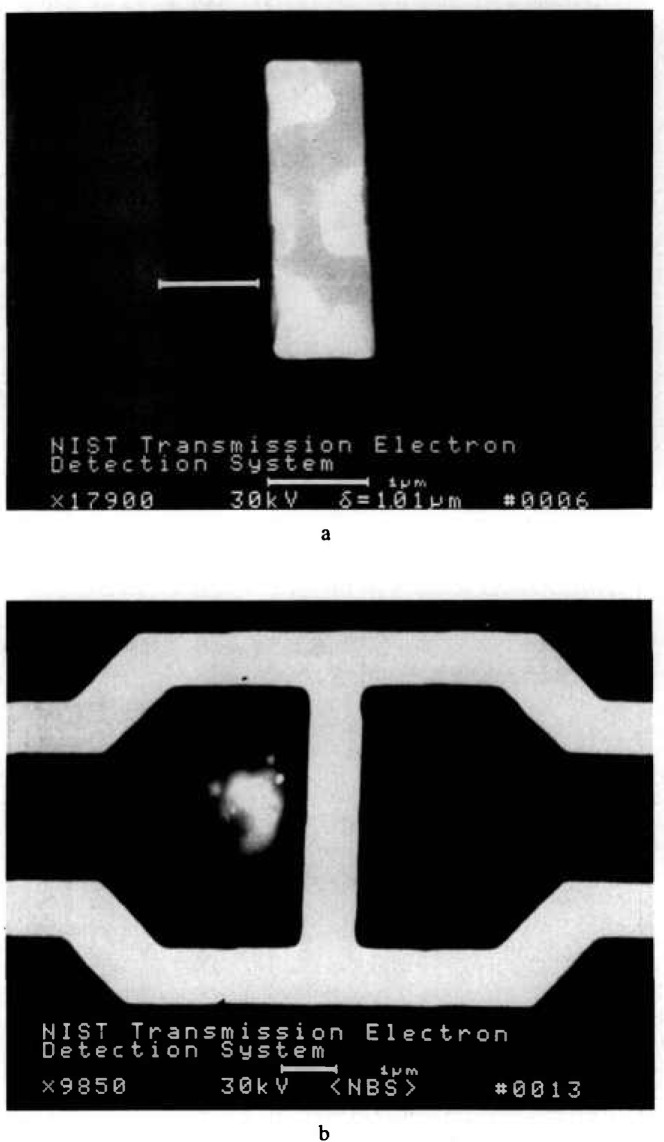
TED mode as applied to x-ray mask repair, (a) Micrograph with intentionally ion-beam-milled windows positioned 1 micrometer from the edge using the Micron ion beam mask repair system, (b) Defect in the absorber to be repaired.

The high contrast image obtained in this mode of electron detection lends itself to less ambiguity regarding the location of the structures of interest. Figure 29a shows a low magnification image of a test pattern, and Fig. 29b displays the pattern at higher magnification, showing the presence of an electron-dense particle. Such dense particles could result in defects on the exposed wafer. Inspection in the secondary electron imaging mode could tend to exaggerate the importance of such a small contaminant particle because of enhanced topographic contrast or particle contrast. In secondary electron detection, a low atomic weight contaminant such as a carbonaceous or siliceous particle could appear bright and thus be misinterpreted as being of a higher atomic number (i.e., a particle of gold) yet, in actuality be relatively transparent to the x rays. The TSEM technique described here yields an image of the x-ray mask similar to the view the wafer has of the x rays passing through the mask during exposure. Overlay comparison of this image in a die-to-database or die-to-standard image could readily detect such particles by high-speed computer systems and image analysis. Another area where the TSEM technique has proven useful has been in x-ray mask repair. Fig. 30 shows micrographs of x-ray masks that have been intentionally modified by focused ion beam milling showing that this technique can be used to find and view mask defects prior to their repair as well as inspect the actual repair work.

## 4. Conclusions

This work has shown that, given the appropriate specimen, novel approaches to metrology issues can result from using the SEM. One appropriate specimen is an x-ray mask which allows the use of the TSEM mode for accurate linewidth metrology. Although not the primary motivation of this work, this technique also lends itself to high-speed x-ray mask defect inspection. Secondary electron imaging is unable to detect large voids within the absorber (even if they are large enough to affect x-ray absorption), whereas these voids could be observed as contrast variations in the transmission electron image. Furthermore, in transparent areas of the mask, secondary electron detection tends to exaggerate the importance of a small contaminant particles because of enhanced topographic contrast or particle contrast. In secondary electron detection, a low atomic weight contaminant such as a carbonaceous or siliceous particle could appear bright and thus be misinterpreted as being of a higher atomic number (i.e., a particle of gold) yet, in actuality be relatively transparent to the x rays. The technique described here yields a clear image of the x-ray mask similar to the view the wafer has of the x rays passing through the mask during exposure. Unfortunately, metrology based on the transmitted-electron image is not readily adaptable to totally opaque specimens such as photoresist on silicon wafers where the need for standards and precise measurements is presently the greatest. However, experiments currently underway can elucidate this problem further. Another question that can only be answered by further experimentation is the magnitude of process bias or the difference between nominal and actual structure that will exist in the final product once an x-ray mask is measured in the SEM and then is used for exposing wafers with the X rays. In addition, since no standards currently exist for the following measurement of the thick layer structures present on the processed wafer, accurate critical dimension verification is not possible.

One advantage of the present TSEM mode for dimensional metrology is its relative insensitivity to some of the major contributors to imprecision of dimensional measurements in the more conventional SEM and TEM modes. Clearly, the axial alignment of the broad-area TSEM detector and the detector-feature spacing are not critical and thereby make the mode more robust to reasonable amounts of misalignment. The broad-area type detector developed for this work also collects more electrons than the narrow-angle type detector and thus improves the signal-to-noise ratio. The signal-to-noise ratio is further enhanced by the large number of data points taken by the laser interferometer system and the averaging of the data points for the same location.

Contamination of the sample by the electron beam is always a potential problem in the SEM, but due to the high beam energies required for the TSEM technique, contamination is not a first order metrology problem in this instance, because any reasonable amount of low atomic-number contamination (carbon, for example) is easily penetrated by the beam electrons (and x rays). However, it should be noted that contamination build-up over time can cause measurement imprecision because of contamination build-up at the base of the absorber which broadens the profile and can also attenuate the signal thus altering the threshold point of the measurement.

The ability to use a high accelerating voltage for the TSEM technique permits the use of smaller beam diameters and thus higher resolution than is possible with low accelerating-voltage instruments. Therefore, as long as the resolution is better than the horizontal projection of the sloping trapezoidal edges of the features being measured, the notch in the transmission that occurs along the edge is theoretically observable. However, in order to make the notch observable in practice it may be necessary to improve: 1) the signal-to-noise ratio by longer observation times at each pixel point of the line profile, 2) improve the smoothness of the sloping edges of the specimen, and/or 3) improve the alignment of the specimen normal to the SEM column axis. Therefore, given these potential advantages with no overriding disadvantages, the expectation is that the TSEM mode can be made as precise, or more precise, than conventional SEM modes of operation with the added advantage afforded by the modeling, which is accuracy.

The major components of potential inaccuracy in contemporary SEM metrology are: 1) imprecision; 2) inability to determine the location of the edge; 3) inadequately calibrated magnification and/or nonlinearity in beam-scanned instruments (or its equivalent in interferometric-stage instruments), and 4) rough edge geometries that not only tend to obscure observation of the notch in the TED mode, but also cloud the very definition of linewidth for any mode of operation.

Edge roughness should be considered an undesirable attribute of the sample, and not necessarily a fundamental metrology problem with the TSEM technique. The treatment of roughness used in this paper should be considered as only a first approximation because the justification for treating the roughness as being equivalent to an added component of effective smooth trapezoidal slope is almost totally phenomenological. The “linewidth” in this approximation was defined to be the average of two calculated linewidths determined by two different methods: 1) by using the theoretical notch positions for a smooth-edge trapezoidal specimen with the measured (in an SEM) geometrical edge slope, and 2) by using the notch positions determined theoretically for smooth edges, but with a larger phenomenological slope that gave better all-over agreement with the experimentally observed profiles. Fortunately, the two “linewidths” so determined were not too different (less than or equal to 10 nm for the 0.25 μm width line). This linewidth difference is clearly a component of systematic inaccuracy of this approximation. A better approach would be to actually model the effects of edge roughness and thereby calculate a more accurate notch position in the presence of edge roughness.

Uncertainty in the measurement process is the combination of the two conceptually different quantities: imprecision and inaccuracy. A conservative approach to uncertainty (i.e., a possible overestimate) will be taken because there is no guarantee that all sources of imprecision and inaccuracy have been identified or were under control. This conservative approach is to simply algebraically add the observed 3 standard deviation imprecision of the measurement to the above quoted value of inaccuracy. This results in an uncertainty of 10 nm for 0.25 μm wide lines because the standard deviation is negligible (see Table 2). The uncertainty in other measurements may be more or less than these values because of different specimens, different SEMs, and the use of different methods of calculating uncertainty. However, the present values of uncertainty do indicate the general magnitude of the uncertainties one might expect to achieve by using the TSEM technique on actual x-ray masks, and that is all that was intended. There remain several issues that should be studied to improve this work even further. One major issue is the development and modification of the computer model to include the edge rounding and surface roughness. A second area of study would be the comparison of the secondary, backscattered and transmitted electron images. This should also be done to evaluate the potential of more conventional SEM modes for x-ray mask metrology. The handling of the data and automatic measurement algorithms are another area that can now be improved. Currently it takes about 4 h to obtain one data set composed of pitch and width data of one nominal line size in both the X and Y directions.

The measurement of x-ray masks with the TED mode presents a unique opportunity to obtain precise and, ultimately, accurate measurements of these samples. This “opens the door” for the development and issuance of NIST traceable standards. This also provides the x-ray lithography community with the only calibrated SEM linewidth standard.

## Figures and Tables

**Table 1:** Gold absorber edge location

Nominal line (μm)	Edge location (%)
0.25	56.85
0.35	58.35
0.50	60.60
0.70	61.55
0.75	61.60
1.00	61.75

**Table 2:** X-ray mask measurements

Nominal line (μm)	Actual measurement (μm)	Standard deviation (μm)
0.25	0.237	0.00015
0.35	0.363	0.00070
0.50	0.487	0.00280
0.75	0.740	0.00070

## Footnotes

1 Certain commercial equipment, instruments, or materials are identified in this paper to specify adequately the experimental procedure. Such identification does not imply recommendation or endorsement by the National Institute of Standards and Technology, nor does it imply that the materials or equipment identified are necessarily the best available for the purpose.
